# Druggable β-catenin palmitoyl-switch coordinates immune evasion via immunogenic ferroptosis resistance and PD-L1-mediated immunosuppression

**DOI:** 10.1016/j.xcrm.2026.102837

**Published:** 2026-05-28

**Authors:** Qiang Zhang, Yue Kong, Yinglin Long, Xue Li, Liang Wang, Zhanhao Luo, Xiaoya Yang, Yanchun Xie, Quanwei Yu, Jing Yu, Dayi Liang, Wenhao Yuan, Xiaomin Cheng, Yaqi Su, Kaisa Cui, Guobao Tian, Zhen He, Ping Lan

**Affiliations:** 1 Zhongshan School of Medicine, Sun Yat-sen University Shenzhen Campus, Shenzhen, Guangdong 518107, China; 2 Department of General Surgery, The Sixth Affiliated Hospital, Sun Yat-sen University, Guangzhou, Guangdong 510655, China; 3 Department of Dermatology, The Second Affiliated Hospital of Guangzhou Medical University, Guangzhou, Guangdong 510260, China; 4 Department of Ultrasound, Guangdong Key Laboratory of Liver Disease Research, the Third Affiliated Hospital of Sun Yat-sen University, Guangzhou, Guangdong 510655, China; 5 Guangdong Provincial Key Laboratory of Colorectal and Pelvic Floor Diseases, Guangdong Institute of Gastroenterology, The Sixth Affiliated Hospital, Sun Yat-sen University, Guangzhou, Guangdong 510655, China; 6 Heilongjiang Institute for Drug Control, Harbin 150088, China; 7 Department of General Surgery, The First Affiliated Hospital, Jiangxi Medical College, Nanchang University, Nanchang, Jiangxi 330006, China; 8 Wuxi Cancer Institute, Affiliated Hospital of Jiangnan University, Wuxi, Jiangsu 214062, China; 9 Key Laboratory of Human Microbiome and Chronic Diseases (Sun Yat-sen University), Ministry of Education, Guangzhou, Guangdong 510655, China; 10 Biomedical Innovation Center, The Sixth Affiliated Hospital, Sun Yat-sen University, Guangzhou, Guangdong 510655, China; 11 State Key Laboratory of Oncology in South China, Guangzhou, Guangdong 510655, China

**Keywords:** β-catenin, palmitoylation, S-nitrosylation, ZDHHC5, immunogenic ferroptosis, SLC7A11, PD-L1, immunosuppression, inhibitor, colorectal cancer

## Abstract

Immune evasion remains a major challenge in colorectal cancer (CRC) treatment. Here, we identify that druggable β-catenin palmitoylation at cysteine 466 (C466) by ZDHHC5 acts as a competitive switch, displacing S-nitrosylation and coordinating an immunosuppressive program. The palmitoylated state stabilizes the β-catenin/TCF4 complex, which simultaneously upregulates SLC7A11 to suppress immunogenic ferroptosis, critical for CD8^+^ T cell priming, and PD-L1 to inhibit cytotoxic CD8^+^ T cells, thereby impairing both the initiation and effector phases of anti-tumor immunity. Clinically, *ZDHHC5* expression predicts poor survival and resistance to anti-PD-L1 therapy. Silencing *ZDHHC5* reduced tumor growth in patient-derived organoids and xenografts, while *Zdhhc5* ablation in mice inhibited CRC progression by reversing immunogenic ferroptosis suppression and alleviating PD-L1-mediated T cell inhibition. Importantly, β-cat-oxazole, a first-in-class inhibitor, disrupts ZDHHC5-β-catenin binding, thereby restoring immunogenic ferroptosis and blocking PD-L1-mediated immunosuppression, ultimately suppressing tumor growth. Our findings establish targeting of this palmitoyl-switch as a precision strategy to overcome therapeutic resistance.

## Highlights

• β-catenin C466 palmitoylation by ZDHHC5 displaces the inhibitory S-nitrosylation
• It inhibits immunogenic ferroptosis and upregulates PD-L1 to drive immune evasion
• Genetic ablation of *Zdhhc5* suppresses colorectal tumorigenesis *in vivo*
• β-cat-oxazole targeting β-catenin C466 palmitoylation activates anti-tumor immunity

## Graphical abstract

**Figure:**
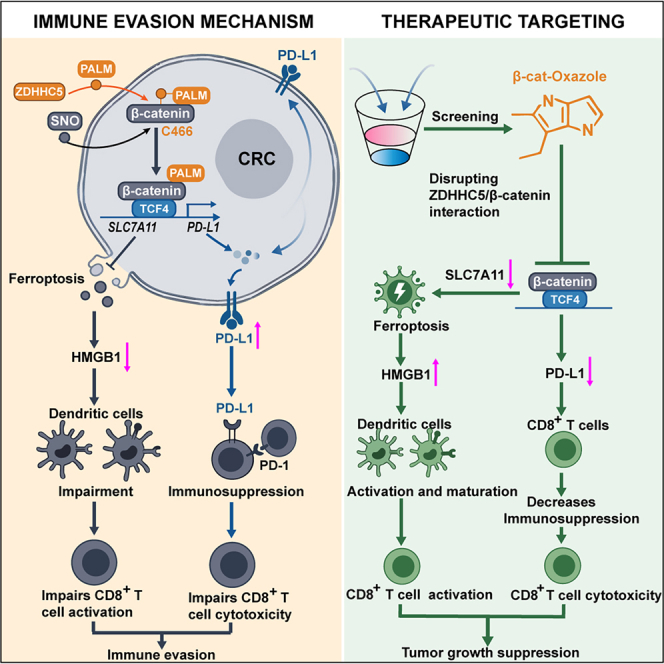

## Teaser

Zhang et al. identify a druggable palmitoylation switch on β-catenin at C466, mediated by ZDHHC5, which displaces inhibitory S-nitrosylation. This switch coordinates immune evasion in colorectal cancer by upregulating SLC7A11 and PD-L1 to block immunogenic ferroptosis and inhibit T cells, and pharmacological inhibition reactivates anti-tumor immunity and suppresses tumor growth.

## Introduction

Immune evasion in colorectal cancer (CRC) presents a significant clinical challenge, particularly as the mechanisms underlying concurrent immunosuppressive pathways remain inadequately understood. Identifying the key regulatory nodes that integrate various immune evasion strategies employed by tumor cells is crucial for enhancing therapeutic efficacy.

The Wnt/β-catenin pathway has emerged as a central player in this context, serving not only as an oncogenic driver but also as a regulator of immune evasion.^1^ This pathway is hyperactivated in over 90% of CRC cases, promoting tumor cell proliferation, stemness, and resistance to therapies.^2^^,^^3^ Importantly, compelling evidence links hyperactivation of the Wnt/β-catenin pathway to both ferroptosis resistance^4^ and immune evasion^1^—two major barriers impeding effective immunotherapy.

The functional diversity of β-catenin in cancer is profoundly shaped by post-translational modifications (PTMs).^5^^,^^6^^,^^7^^,^^8^^,^^9^ Our prior work identified palmitoylation at β-catenin cysteine 466 (C466) as a critical oncogenic regulator that stabilizes β-catenin and enhances its transcriptional activity in CRC,^10^ supported by mass spectrometry (MS) analyses conducted by others confirming the presence of palmitoylation at this site.^11^^,^^12^ Interestingly, C466 is also a target for S-nitrosylation, which inhibits β-catenin’s transcriptional function.^13^ The interplay between these competitive PTMs may contribute to cancer pathogenesis, challenging the conventional understanding of PTMs as isolated events.

Despite these advances, two critical gaps persist. (1) The specific palmitoyltransferase responsible for modulating β-catenin at C466 has yet to be identified. Although ZDHHC9 has been shown to palmitoylate β-catenin at C300 and promote its degradation in the context of renal fibrosis,^14^ this mechanism appears inconsistent with ZDHHC9’s established oncogenic role in various cancers,^15^^,^^16^^,^^17^^,^^18^^,^^19^^,^^20^ including CRC.^15^ This discrepancy suggests that ZDHHC9-mediated β-catenin palmitoylation at C300 may not be the primary mechanism driving its pro-tumorigenic functions in cancer. Consequently, identifying the enzyme that catalyzes the oncogenic C466 palmitoylation of β-catenin in CRC is of critical importance. (2) The specific biological consequences of β-catenin C466 palmitoylation, particularly its role in coordinating immunosuppressive outputs, remain to be defined. Given the association of hyperactivated Wnt/β-catenin signaling with both ferroptosis resistance^4^ and immune evasion,^1^ we hypothesize that palmitoylation at C466 of β-catenin could integrate these resistance mechanisms.

Emerging evidence suggests that resistance to ferroptosis inhibits immunogenic cell death, thereby preventing the release of damage-associated molecular patterns (DAMPs), such as high-mobility group box 1 (HMGB1). HMGB1 is a crucial alarmin that activates dendritic cells (DCs) and promotes antigen presentation, which is essential for initiating CD8^+^ T cell-mediated anti-tumor responses.^21^ Furthermore, oncogenic β-catenin transcription factors can enhance the expression of immune checkpoints like PD-L1.^22^ Thus, we hypothesize that palmitoylation of β-catenin at C466 may simultaneously obstruct immunogenic ferroptosis (e.g., through DAMP release inhibition) and drive PD-L1-mediated immune evasion. Validating this hypothesis could reveal a valuable therapeutic target.

Our study aims to (1) identify the enzyme responsible for directing palmitoylation of β-catenin at C466 in CRC, (2) determine whether β-catenin C466 palmitoylation facilitates immune suppression by coordinating resistance to immunogenic ferroptosis and immune checkpoint activation, and (3) validate its druggability through selective inhibition. If substantiated, competitive palmitoylation at C466 may replace S-nitrosylation, positioning β-catenin as a critical mediator that simultaneously suppresses immunogenic ferroptosis and promotes PD-L1-mediated immune evasion, thus uncovering a therapeutically relevant vulnerability.

## Results

### ZDHHC5 interacts with β-catenin

**Figure 1:**
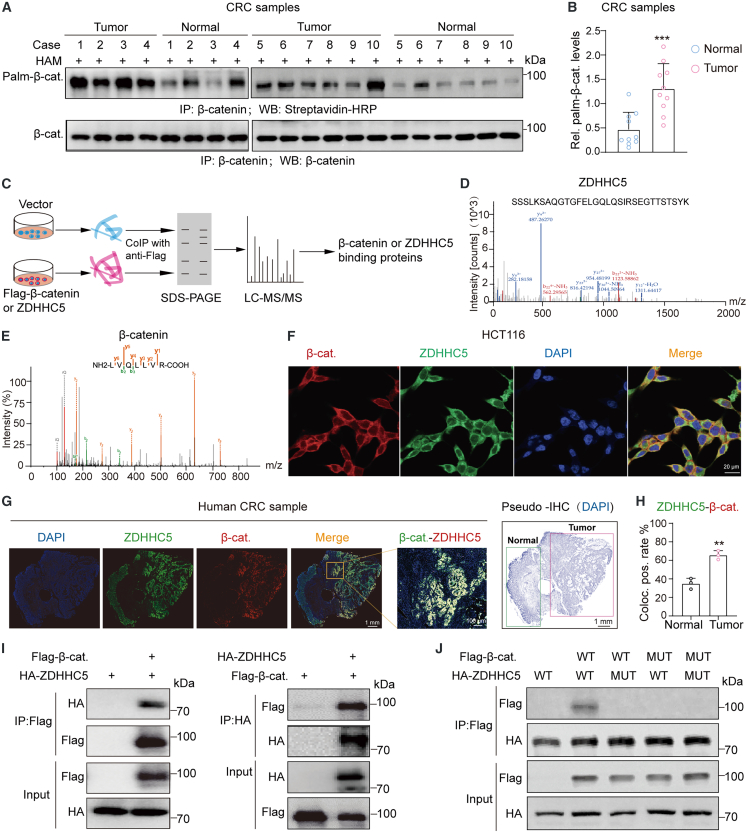
ZDHHC5 interacts with β-catenin (A) Detection of β-catenin palmitoylation in human CRC tissues. Paired tumor and adjacent normal tissues from 10 CRC patients were subjected to acyl-biotin exchange (ABE) assays. (B) Quantification of palmitoylated β-catenin levels shown in (A). (C) Experimental workflow for identifying β-catenin or ZDHHC5 interactors. FLAG-tagged β-catenin or ZDHHC5 was expressed in HEK293T cells, immunoprecipitated, and subjected to MS. (D and E) Reciprocal IP/MS identifies a potential ZDHHC5-β-catenin interaction. MS spectra demonstrate the mutual enrichment of ZDHHC5 peptides in FLAG-β-catenin immunoprecipitates (D) and β-catenin peptides in FLAG-ZDHHC5 immunoprecipitates (E). (F) Subcellular co-localization of ZDHHC5 and β-catenin in HCT116 cells. Representative immunofluorescence microscopy images of HCT116 cells stained for β-catenin (red), ZDHHC5 (green), and nuclei (DAPI, blue). Individual channels and the merged image are shown. Scale bar, 20 μm. (G and H) ZDHHC5-β-catenin co-localization in human CRC tissues. Representative immunofluorescence images of a human CRC specimen and matched adjacent normal tissue stained for ZDHHC5 (green), β-catenin (red), and nuclei (DAPI, blue) (G). Quantification of the co-localization intensity between ZDHHC5 and β-catenin signals in tumor versus adjacent normal regions (H). Scale bars: 1 mm (overview) and 100 μm (zoom). (I) CoIP analysis of the ZDHHC5-β-catenin interaction. FLAG-β-catenin and hemagglutinin (HA)-ZDHHC5 plasmids were transfected into HEK293T cells, followed by coIP assays. (J) CoIP analysis of the ZDHHC5-β-catenin interaction using interface mutants. CoIP assay was performed in HEK293T cells co-transfected with plasmids encoding FLAG-tagged WT or interface-mutant β-catenin (Val349A, Asn430A, Pro505A, Phe560A, labeled MUT) and HA-tagged WT or interface-mutant ZDHHC5 (Phe23A, Tyr50A, Met161A, Val192A, Leu195A, Phe196A, Phe197A, Ile198A, Gly205A, Phe206A, Val208A, Val209A, labeled MUT), as indicated. Data are presented as mean ± SD; statistical significance was determined by Student’s *t* test; ∗∗*p* < 0.01, ∗∗∗*p* < 0.001.

Our previous work established that palmitoylation at C466 of β-catenin drives CRC progression,^10^ a finding supported by elevated β-catenin palmitoylation in CRC versus normal tissues (Figures 1A and 1B). To identify the responsible enzyme, we conducted immunoprecipitation of FLAG-β-catenin from HEK293T cells, followed by MS, which identified ZDHHC5 as a potential interactor (Figures 1C, 1D, and S1A). Reciprocal MS with FLAG-ZDHHC5 confirmed β-catenin binding (Figures 1C, 1E, and S1B).

We next sought to experimentally validate this interaction in CRC models. Immunofluorescence showed pronounced co-localization of ZDHHC5 and β-catenin in HCT116 and DLD1 cell lines (Figures 1F and S1C) and clinical specimens, with stronger co-localization in tumors than matched normal tissue (Figures 1G and 1H). Co-immunoprecipitation (coIP) in HEK293T cells further confirmed the physical interaction (Figure 1I).

To define the interaction interface at the molecular level, we generated a predicted complex structure using AlphaFold3, which suggested complementary interfaces between ZDHHC5 and β-catenin (Figures S1D and S1E). We then employed a structure-guided mutagenesis strategy to test the functional relevance of this predicted interface. Molecular dynamics simulation indicated this predicted complex remained stable over 20 ns (root-mean-square deviation convergence; Figure S1F). Residue decomposition analysis of this simulation identified potential contact residues on both proteins, including residues from ZDHHC5 (e.g., Ile198 and Phe206) and β-catenin (e.g., Val349 and Phe560) (Figures S2A and S2B). Critically, mutation of these predicted key residues completely abrogated the ZDHHC5-β-catenin interaction in coIP assays (Figure 1J). Collectively, these results establish an interaction between ZDHHC5 and β-catenin.

### ZDHHC5 palmitoylates β-catenin at C466

**Figure 2:**
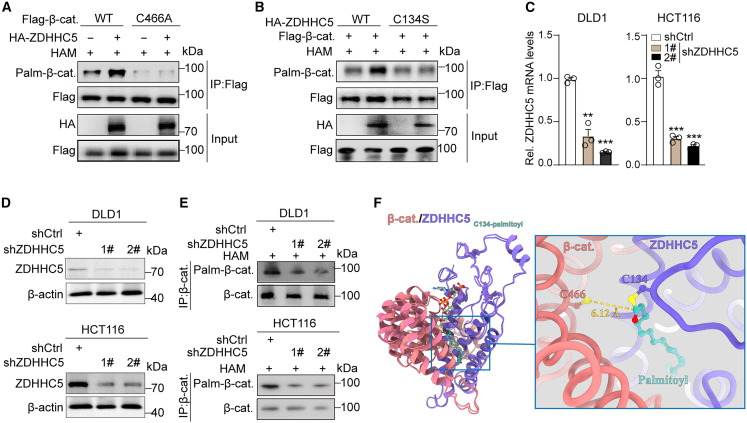
ZDHHC5 palmitoylates β-catenin at C466 (A) Site-specific palmitoylation of β-catenin at C466 by ZDHHC5. Co-expression of WT β-catenin or its C466A mutant with ZDHHC5 in HEK293T cells, followed by ABE assays. (B) ZDHHC5-mediated palmitoylation of β-catenin is dependent on ZDHHC5 enzyme activity. Co-expression of WT ZDHHC5 or its enzyme-inactive mutant C134S with β-catenin in HEK293T cells, followed by ABE assays. (C and D) Validation of stable *ZDHHC5* knockdown in CRC cell lines. Stable *ZDHHC5* knockdown was established in DLD1 and HCT116 cells, followed by qPCR (C) and immunoblot (D) analysis. (E) Knockdown of *ZDHHC5* reduces β-catenin palmitoylation. Stable *ZDHHC5* knockdown was established in DLD1 and HCT116 cells, followed by ABE assays. (F) Predicted catalytic interface between ZDHHC5 and β-catenin. (Left) Overall structure of the predicted ZDHHC5 (purple)-β-catenin (pink) complex, generated by computational modeling. The boxed region indicates the interface shown in the close-up view. (Right) Close-up of the predicted catalytic site. The auto-acylated catalytic cysteine of ZDHHC5 (C134, blue) and the substrate cysteine of β-catenin (C466, red) are highlighted. A palmitoyl moiety (cyan sticks) is modeled on C134, representing the acyl-enzyme intermediate. The Cγ(C134)-Sγ(C466) distance is ∼6.12 Å (dashed line). Data are presented as mean ± SD; statistical significance was determined by Student’s *t* test; ∗∗*p* < 0.01, ∗∗∗*p* < 0.001.

To explore whether ZDHHC5 palmitoylates β-catenin, we co-expressed ZDHHC5 with either wild-type (WT) β-catenin or the C466A mutant in HEK293T cells. Acyl-biotin exchange assays revealed that ZDHHC5 overexpression increased the palmitoylation level of WT β-catenin, but not the C466A mutant (Figure 2A), thereby confirming the specificity of ZDHHC5-mediated palmitoylation at the C466 site. The catalytically inactive ZDHHC5 C134S mutant^23^ completely abolished the palmitoylation of WT β-catenin (Figure 2B), establishing the requirement for ZDHHC5’s enzymatic function. Furthermore, stable knockdown of *ZDHHC5* in HCT116 and DLD1 CRC cell lines (Figures 2C and 2D) significantly reduced endogenous β-catenin palmitoylation (Figure 2E). These experiments demonstrate that ZDHHC5 is necessary and sufficient for site-specific palmitoylation of β-catenin at C466.

To assess the structural plausibility of this catalytic relationship—given that palmitoylation typically involves the transfer of a palmitoyl moiety from the auto-acylated active-site cysteine of a ZDHHC enzyme to the target cysteine^24^^,^^25^^,^^26^—we analyzed a predicted complex model. This *in silico* analysis suggested that the distance between the carbonyl carbon of acylated C134 in ZDHHC5 and the sulfur of C466 in β-catenin is approximately 6.12 Å (Figure 2F). This predicted proximity is consistent with a spatially feasible catalytic transfer, considering the flexible loop that often harbors the ZDHHC active site.^27^ We emphasize that this model provides a mechanistic hypothesis consistent with our functional data.

Collectively, the combination of genetic, biochemical, and cell-based evidence establishes ZDHHC5 as the enzyme responsible for site-specific palmitoylation of β-catenin at C466, with computational modeling offering a plausible structural context for this interaction.

### Competitive palmitoylation at C466 displaces S-nitrosylation to enhance β-catenin transcriptional activity

**Figure 3:**
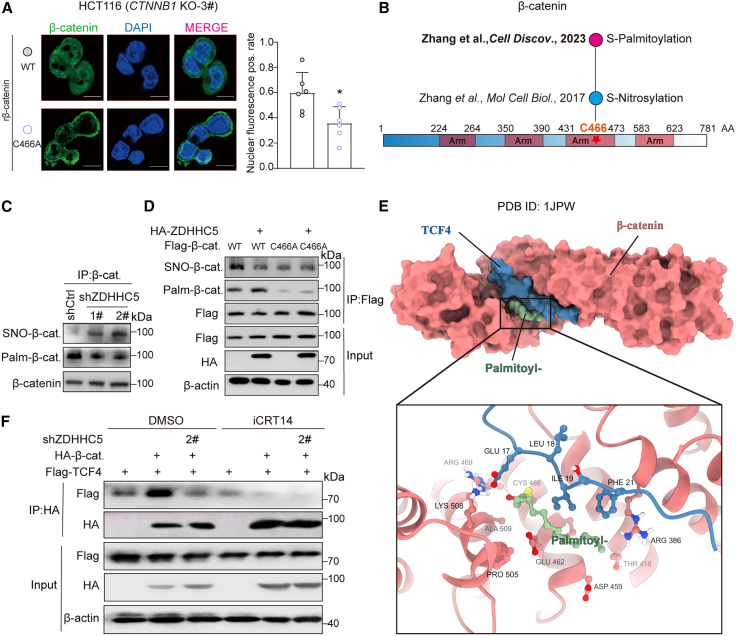
Competitive palmitoylation at C466 displaces S-nitrosylation to potentiate β-catenin transcriptional activity (A) Nuclear accumulation of β-catenin requires palmitoylation at C466. WT β-catenin or its C466A mutant was re-expressed in *CTNNB1*-knockout HCT116 cells, followed by immunofluorescence assays (left). Quantification of nuclear β-catenin intensity (right). Scale bars, 10 μm. (B) Schematic of competitive PTM crosstalk. Model depicting palmitoylation and S-nitrosylation competition at β-catenin C466. (C) β-Catenin palmitoylation inhibits its S-nitrosylation. Stable *ZDHHC5* knockdown in HEK293T cells was performed, and the cells were evenly divided into two groups. One group underwent ABE analysis, and the other group was subjected to S-nitrosylation detection. (D) Palmitoylation of β-catenin at C466 inhibits S-nitrosylation at the same site. ZDHHC5 was co-expressed with WT β-catenin or C466A mutant in HEK293T cells, followed by ABE and S-nitrosylation detection assays. (E) A computational model of the β-catenin/TCF4 complex with palmitoylation modeled at β-catenin C466. The structural model is derived from the experimental co-crystal structure (PDB ID: 1JPW). (Top) Surface representation of the overall complex, with β-catenin in blue and TCF4 in orange. A black box highlights the location of the C466 residue. (Bottom) Close-up view of the C466 site. The palmitoyl moiety (green sticks) was modeled onto CYS 466 (yellow sticks) and is shown occupying a pocket at the β-catenin/TCF4 interface. Key surrounding amino acid residues from β-catenin (e.g., LYS 508) and TCF4 (e.g., GLU 17, PHE 21) are displayed as sticks. See Figure S4 for details. (F) CoIP analysis of the β-catenin/TCF4 interaction following *ZDHHC5* knockdown. HA-β-catenin, FLAG-TCF4, and shZDHHC5 were co-expressed into HEK293T cells, which were treated with DMSO or iCRT4 (100 μM), followed by coIP assays. Data are presented as mean ± SD; statistical significance was determined by Student’s *t* test; ∗*p* < 0.05.

Our prior work has identified C466 palmitoylation as a positive regulator of β-catenin transcriptional activity.^10^ To assess the function of C466 palmitoylation, we first expressed the C466A mutant in HEK293T cells and observed its impaired nuclear accumulation relative to WT β-catenin (Figure S3A), implicating this modification in β-catenin trafficking. To definitively test this in a clean genetic background, we turned to *CTNNB1*-knockout HCT116 cells (Figure S3B). Reconstitution with the C466A mutant again resulted in markedly reduced nuclear β-catenin compared to WT-reconstituted cells (Figure 3A). Furthermore, transcriptomic analyses revealed significant downregulation of Wnt/β-catenin signaling upon *ZDHHC5* knockdown in DLD1 cells (Figure S3C). This was accompanied by a consistent suppression of β-catenin target genes in both DLD1 and HCT116 cells (Figure S3D), thereby supporting the conclusion that C466 palmitoylation is critical for β-catenin’s nuclear function.

Interestingly, C466 also serves as a site for inhibitory S-nitrosylation, which disrupts the formation of the β-catenin/TCF4 complex and diminishes transcriptional output.^13^ We hypothesized that palmitoylation at this residue could competitively displace S-nitrosylation (Figure 3B). Supporting this hypothesis, we observed that *ZDHHC5* knockdown resulted in increased β-catenin S-nitrosylation in HEK293T cells (Figure 3C). Conversely, ZDHHC5 overexpression led to a reduction in S-nitrosylation levels; this effect was abolished when β-catenin C466A was expressed (Figure 3D), indicating a direct competition between these PTMs at C466.

To investigate a potential mechanism by which C466 palmitoylation could enhance β-catenin transcriptional activity, we generated a structural hypothesis. Based on the β-catenin/TCF4 co-crystal structure, *in silico* modeling of a palmitoyl moiety at C466 suggested a model in which the palmitate occupies a hydrophobic pocket (Figure 3E). This palmitoyl occupancy is predicted to allosterically stabilize a conformation that enables intermolecular interactions, most notably a salt bridge between β-catenin Lys508 and TCF4 Glu17, and new hydrogen bonds involving β-catenin Asp390/Lys354 and TCF4 Phe21 (Figures S4A and S4B). This model provided a testable mechanistic hypothesis for increased affinity.

To experimentally test the key prediction of this hypothesis—that C466 palmitoylation strengthens the β-catenin/TCF4 interaction—we performed coIP assays. As predicted, *ZDHHC5* knockdown significantly weakened the β-catenin-TCF4 interaction in HEK293T cells (Figure 3F). To rule out the possibility that this effect resulted from nonspecific disruption of the complex, we employed iCRT14, a small-molecule inhibitor that competitively occupies the TCF4-binding interface on β-catenin.^28^ In the presence of iCRT14, which nearly abolished the baseline interaction, *ZDHHC5* knockdown no longer produced an additional inhibitory effect (Figure 3F). This confirms that ZDHHC5 modulates the intrinsic binding affinity at the canonical interface, rather than causing nonspecific complex destabilization. Collectively, these findings establish a PTM competition paradigm wherein palmitoylation at C466 displaces inhibitory S-nitrosylation, thereby stabilizing the β-catenin-TCF4 complex and amplifying its transcriptional output.

### β-catenin palmitoylation at C466 confers immunogenic ferroptosis resistance through *SLC7A11* transcriptional activation

**Figure 4:**
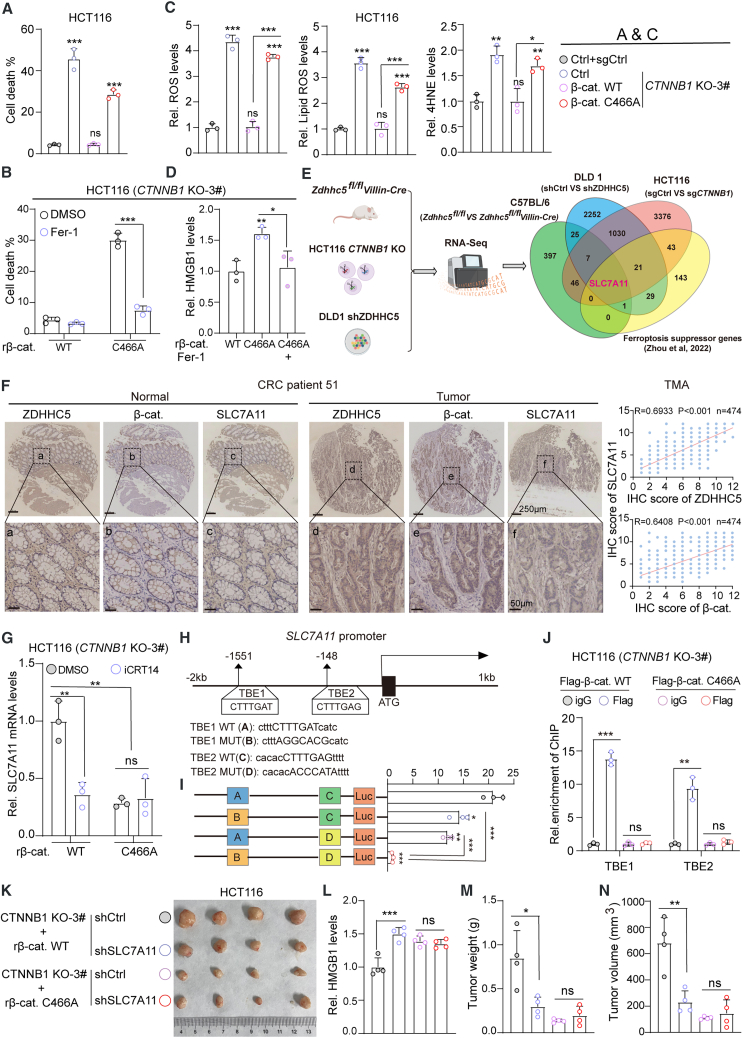
β-catenin palmitoylation at C466 confers immunogenic ferroptosis resistance through *SLC7A11* transcriptional activation (A and B) Cell death in *CTNNB1*-knockout HCT116 cells reconstituted with WT or C466A β-catenin, with or without ferroptosis inhibition. *CTNNB1*-knockout HCT116 cells were reconstituted with WT β-catenin (β-cat. WT) or palmitoylation-deficient C466A mutant (β-cat. C466A). In (A), cells were left untreated, while in (B), cells were treated with ferrostatin-1 (10 μM). Cell death was assessed by trypan blue staining. (C) Analysis of ferroptosis markers in *CTNNB1*-knockout HCT116 cells reconstituted with β-catenin variants. In *CTNNB1*-knockout HCT116 cells, β-catenin WT or the C466A mutant was re-expressed, and the corresponding markers were measured. (D) Relative HMGB1 release levels in β-catenin-reconstituted *CTNNB1*-knockout HCT116 cells. HMGB1 levels in the culture supernatant were measured by ELISA following re-expression of the indicated β-catenin variants in *CTNNB1*-knockout HCT116 cells and treatment with or without ferrostatin-1 (10 μM). (E) Integrated transcriptomic analysis identifies SLC7A11 as a common target of the ZDHHC5/β-catenin axis. Integrated transcriptomics of intestinal epithelial-specific *Zdhhc5*-knockout mice, *CTNNB1*-knockout HCT116 cells, and *ZDHHC5*-knockdown DLD1 cells identified downregulated genes, which were then analyzed for overlap with previously reported ferroptosis-related genes. (F) Immunohistochemistry (IHC) analysis and correlation of ZDHHC5, β-catenin, and SLC7A11 in CRC TMA. Representative IHC images for the expression of ZDHHC5, β-catenin, and SLC7A11 in CRC TMA (left). Correlation analysis of SLC7A11 with ZDHHC5 and β-catenin in CRC TMA (right). Scale bars: 250 μm (overview) and 50 μm (magnified views). (G) Regulation of *SLC7A11* transcription by β-catenin C466 palmitoylation via the β-catenin/TCF4 complex. *CTNNB1*-knockout HCT116 cells were reconstituted with either WT β-catenin or the C466A mutant and treated with iCRT14 (100 μM) for 24 h, followed by qPCR analysis. (H) Potential β-catenin/TCF4 binding sites on the *SLC7A11* locus. (I) β-catenin binds TBEs in the *SLC7A11* promoter. Luciferase assays were performed to evaluate the activity of different SLC7A11 promoter constructs in HEK293T cells expressing exogenous β-catenin. (J) Chromatin occupancy of β-catenin at TCF/LEF-binding elements (TBEs) of the SLC7A11 locus is dependent on C466 palmitoylation. In *CTNNB1*-knockout HCT116 cells, WT β-catenin or the C466A mutant was re-expressed, followed by chromatin immunoprecipitation analysis. (K) β-catenin C466 palmitoylation-dependent SLC7A11 promotes tumor growth. *CTNNB1*-knockout HCT116 cells were re-expressed with WT β-catenin or its C466A mutant and subcutaneously injected into nude mice. Tumor growth was monitored, and after reaching a certain size, tumors were treated with lentivirus-shSLC7A11 injections. (L) Relative levels of HMGB1 in plasma from BALB/c nude mice bearing HCT116-derived xenografts shown in (K), measured using a human HMGB1 ELISA kit. (M and N) Quantification of tumor weight (M) and tumor volume (N) from (K). Data are presented as mean ± SD; statistical significance was determined by Student’s *t* test; ∗*p* < 0.05, ∗∗*p* < 0.01, ∗∗∗*p* < 0.001; ns, not significant.

β-catenin signaling is critical for tumor cell survival.^29^^,^^30^ To investigate whether palmitoylation at C466 modulates sensitivity to cell death, we manipulated ZDHHC5 expression in CRC cell lines. Knockdown of *ZDHHC5* resulted in increased cell mortality, which was specifically rescued by the ferroptosis inhibitor ferrostatin-1,^31^ but not by inhibitors of apoptosis (Z-VAD-FMK^32^), necroptosis (necrostatin-1^33^), or autophagy (3-MA^34^) (Figures S5A and S5B). Conversely, overexpression of ZDHHC5 reduced ferroptosis induced by RSL3^35^ and diminished the accumulation of reactive oxygen species (ROS), lipid ROS, and 4-hydroxynonenal (4-HNE) (Figures S5C–S5F). *ZDHHC5* knockdown resulted in elevated levels of ROS and 4-HNE in both HCT116 and DLD1 cells (Figures S5G and S5H). Notably, in *CTNNB1*-knockout HCT116 cells, manipulation of ZDHHC5 failed to alter these ferroptosis markers (Figures S6A–S6G), indicating that β-catenin is required for ZDHHC5’s effect. We next directly tested the functional requirement for palmitoylation at the C466 site. Reconstitution of *CTNNB1*-knockout HCT116 cells with WT β-catenin fully rescued the cell death, consistent with the restoration of ferroptosis resistance (Figure 4A). In contrast, reconstitution with the palmitoylation-deficient C466A mutant provided only a partial rescue (Figure 4A). This partial phenotype suggested that cells expressing the C466A mutant retain heightened intrinsic sensitivity to ferroptosis. To test this, we assessed the protective efficacy of ferrostatin-1. As predicted, ferrostatin-1 conferred significantly greater protection in C466A-reconstituted cells compared to WT-reconstituted controls (Figure 4B). Furthermore, C466A-reconstituted cells consistently exhibited higher levels of ROS, lipid ROS, and 4-HNE than their WT-reconstituted counterparts (Figure 4C). Collectively, these data demonstrate that palmitoylation at C466 is essential for β-catenin to confer resistance to ferroptosis.

Having established that β-catenin palmitoylation at C466 confers ferroptosis resistance, we next investigated its downstream mechanism. Ferroptosis is known to trigger the release of the chromatin-binding protein HMGB1, a key DAMP and immune mediator.^36^ Consistently, we found that HCT116 cells reconstituted with the palmitoylation-deficient β-catenin C466A mutant released significantly more extracellular HMGB1 compared to WT-reconstituted cells (Figure 4D). This enhanced release was blocked by the ferroptosis inhibitor ferrostatin-1 (Figure 4D), confirming its dependence on ferroptotic cell death. To identify the transcriptional target mediating this effect, we performed integrated transcriptomic analyses across multiple models, including intestinal epithelium-specific *Zdhhc5*-knockout mice, *CTNNB1*-knockout HCT116 cells, and *ZDHHC5*-knockdown DLD1 cells. Cross-referencing with a ferroptosis-related gene set^37^ consistently identified SLC7A11, a central inhibitor of ferroptosis, as the top downregulated target (Figure 4E). Clinically, SLC7A11 protein levels positively correlated with both β-catenin and ZDHHC5 in human CRC tissue microarrays (TMA) (Figure 4F), a finding corroborated by public spatial transcriptomics data^38^ (Figures S7A–S7C). Functionally, *ZDHHC5* knockdown decreased *SLC7A11* expression in a β-catenin-dependent manner, and C466A-reconstituted cells exhibited lower SLC7A11 levels than WT-reconstituted cells (Figures S7D–S7F). The β-catenin/TCF4 transcriptional inhibitor iCRT14 suppressed *SLC7A11* expression in WT- but not C466A-reconstituted cells (Figures 4G and S7G), directly linking C466 palmitoylation to β-catenin-dependent *SLC7A11* transactivation.

Mechanistically, we identified two conserved β-catenin/TCF4-binding elements in the *SLC7A11* promoter (Figure 4H). Luciferase reporter and chromatin immunoprecipitation assays confirmed that WT, but not the C466A mutant, β-catenin binds to these sites (Figures 4I. 4J, and S7H), demonstrating that C466 palmitoylation is required for promoter occupancy. To test the *in vivo* relevance and necessity of this SLC7A11-dependent mechanism, we established HCT116-derived xenograft models. *SLC7A11* knockdown potently inhibited tumor growth and increased plasma HMGB1 levels in WT-reconstituted tumors, but had minimal effect in C466A-reconstituted tumors (Figures 4K–4N). Collectively, these data demonstrate that β-catenin palmitoylation at C466 drives *SLC7A11* transcription by facilitating its promoter binding, thereby establishing a molecular pathway that confers resistance to immunogenic ferroptosis and promotes tumor growth.

### β-catenin palmitoylation at C466 coordinates an immunosuppressive program by conferring resistance to immunogenic ferroptosis and upregulating PD-L1

**Figure 5:**
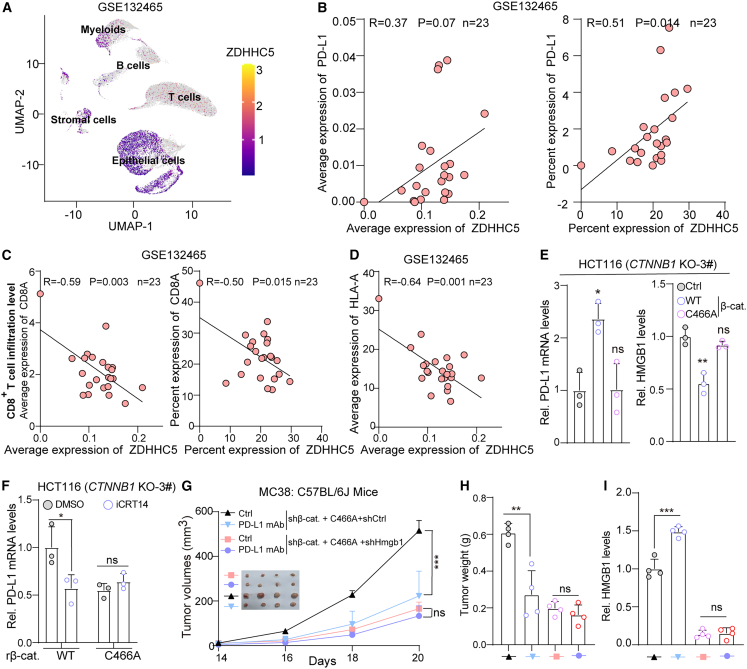
β-catenin palmitoylation at C466 coordinates an immunosuppressive program by conferring resistance to immunogenic ferroptosis and upregulating PD-L1 (A) Uniform manifold approximation and projection plot of CRC single-cell RNA sequencing data (GSE132465), showing the clustering patterns of cells. *ZDHHC5* expression is represented by color intensity, with a scale from low to high. (B) Significant positive correlation between *ZDHHC5* and *PD-L1* expression. Correlation analysis of *ZDHHC5* and *PD-L1* expression at both average (left) and relative (right) expression levels, based on (A). (C) Significant negative correlation between *ZDHHC5* and CD8^+^ T cell infiltration. Correlation analysis of *CD8A* and *ZDHHC5* expression at both average (left) and relative (right) expression levels, based on (A). (D) Significant negative correlation between *ZDHHC5* expression in tumor epithelial cells and *HLA-A* expression in DCs. Correlation analysis was performed using the average expression levels of *ZDHHC5* in tumor epithelial cells and *HLA-A* in DCs, based on (A). (E) β-catenin C466 palmitoylation is crucial for *PD-L1* transactivation and suppression of released HMGB1. *CTNNB1*-knockout HCT116 cells were re-expressed with WT or C466A-mutant β-catenin, followed by qPCR analysis (left) and ELISA of culture supernatants (right). (F) *PD-L1* expression is dependent on the β-catenin/TCF4 complex. *CTNNB1*-knockout HCT116 cells were re-expressed with WT or C466A-mutant β-catenin, treated with iCRT4 (100 μM) for 24 h, and analyzed by qPCR. (G) HMGB1 release is required for the efficacy of PD-L1 blockade in the context of β-catenin signaling suppression. *Ctnnb1*-knockdown MC38 cells reconstituted with C466A β-catenin or both C466A β-catenin and *Hmgb1* knockdown were injected into immunocompetent C57BL/6J mice, and tumor volume was monitored after administration of 100 μg anti-PD-L1 on days 14 and 18. (H) Quantification of tumor weight from (G). (I) Relative levels of HMGB1 in plasma from C57BL/6 mice bearing MC38-derived xenografts shown in (G), measured using a mouse HMGB1 ELISA kit. Data are presented as mean ± SD; statistical significance was determined by Student’s *t* test; ∗*p* < 0.05, ∗∗*p* < 0.01, ∗∗∗*p* < 0.001; ns, not significant.

To define the immunosuppressive function of the ZDHHC5-β-catenin axis, we focused on PD-L1 as a prime candidate effector. This focus was motivated by established reports that the β-catenin/TCF complex can transcriptionally upregulate PD-L1,^22^^,^^39^ coupled with our finding that C466 palmitoylation amplifies β-catenin’s transcriptional activity. We, therefore, first examined the association of this axis with PD-L1 and immune context. Analysis of The Cancer Genome Atlas colon adenocarcinoma data revealed significant positive correlations between *ZDHHC5* and *PD-L1* expression, as well as between *CTNNB1* and *PD-L1* (Figure S8A). We validated and extended these findings using a CRC single-cell RNA-seq dataset GSE132465. This analysis confirmed a positive correlation between *ZDHHC5* and *PD-L1* and further identified a significant negative correlation between *ZDHHC5* levels and CD8^+^ T cell infiltration (Figures 5A–5C). Given the critical role of HMGB1—released during ferroptosis—in DC activation and antigen presentation,^21^ we hypothesized that high *ZDHHC5* might be linked to impaired DC function. Consistent with this, *ZDHHC5* expression showed a strong negative correlation with *HLA-A* levels in tumor-associated DCs, with a similar trend for other *HLA* genes (Figures 5D and S8B). These correlative data position the ZDHHC5-β-catenin axis within an immunosuppressive context featuring impaired antigen presentation and exhausted T cells.

To establish a direct causal relationship, we conducted functional rescue experiments. Reconstitution of *CTNNB1*-knockout HCT116 cells with WT β-catenin significantly induced *PD-L1* expression and inhibited extracellular HMGB1 release (Figure 5E). In contrast, reconstitution with the palmitoylation-deficient C466A mutant failed to elicit either response (Figure 5E). Pharmacological inhibition of β-catenin/TCF4 signaling suppressed *PD-L1* transcription in WT-reconstituted cells, but had no effect in C466A-reconstituted cells (Figure 5F). This demonstrates that C466 palmitoylation is essential for β-catenin/TCF4-dependent transactivation of *PD-L1*.

Our model posits that β-catenin C466 palmitoylation confers resistance by simultaneously suppressing immunogenic ferroptosis (limiting HMGB1 release) and upregulating PD-L1. We, therefore, asked if these two arms are functionally integrated in conferring immunotherapy resistance *in vivo*. We implanted *Ctnnb1*-knockdown MC38 cells, reconstituted with either WT or C466A β-catenin and with or without concurrent *Hmgb1* knockdown, into immunocompetent mice and treated them with anti-PD-L1 antibody. Tumors expressing the C466A mutant (which releases HMGB1) exhibited significantly enhanced sensitivity to anti-PD-L1 therapy (Figures 5G and 5H). This sensitivity was associated with elevated plasma HMGB1 levels (Figure 5I). Critically, the therapeutic benefit of anti-PD-L1 was completely abrogated in tumors with combined β-catenin C466 palmitoylation deficiency and *Hmgb1* knockdown (Figures 5G and 5H). This genetic epistasis experiment demonstrates that the immunogenic signal from ferroptosis (HMGB1 release) is necessary to unlock the efficacy of checkpoint blockade (anti-PD-L1).

Collectively, these results demonstrate that ZDHHC5-mediated β-catenin palmitoylation at C466 drives immune evasion through a unified mechanism. It coordinately suppresses immunogenic ferroptosis (limiting HMGB1 release) while simultaneously enhancing PD-L1-mediated T cell inhibition. Our finding of a negative correlation between *ZDHHC5* and *HLA* gene expression in DCs, together with the established role of HMGB1 in DC activation, supports a model whereby this axis may also compromise antigen presentation. Critically, our *in vivo* epistasis experiment confirms that these two immunosuppressive arms are functionally interdependent; the immunogenic signal HMGB1 is required to unlock the efficacy of checkpoint blockade, together establishing a formidable barrier to immunotherapy.

### *ZDHHC5* deficiency reverses immunosuppression and inhibits colorectal tumorigenesis

**Figure 6:**
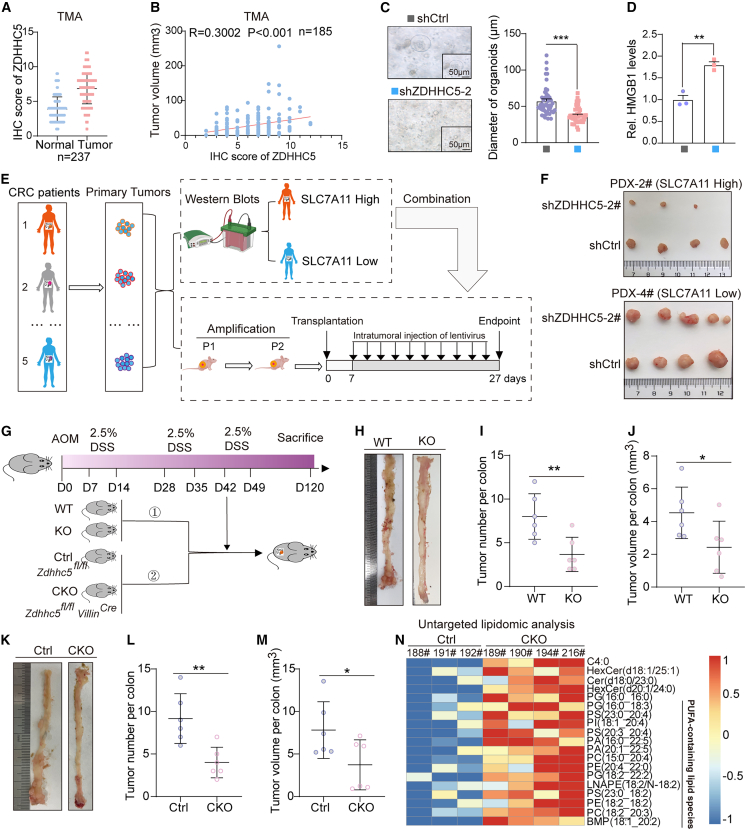
*ZDHHC5* deficiency reverses immunosuppression and inhibits colorectal tumorigenesis (A) IHC analysis of ZDHHC5 expression in tumor tissue compared to normal tissue from CRC TMA. (B) Correlation of ZDHHC5 expression with tumor burden in a TMA cohort of CRC patients. (C and D) Growth and HMGB1 release of CRC patient-derived organoids upon *ZDHHC5*. CRC patient-derived organoids were treated with lentivirus expressing shZDHHC5, and growth was assessed by measuring diameter (C). HMGB1 release was quantified by ELISA of culture supernatants (D). Scale bars, 50 μm. (E) Experimental design for the PDX model. (F) *ZDHHC5* knockdown significantly reduces tumor growth in PDX models with high SLC7A11 expression, but not in low-SLC7A11-expression models. PDXs with high or low SLC7A11 expression were treated with lentivirus expressing shZDHHC5, and tumors were harvested after a period of treatment. (G) Diagram illustrating global *Zdhhc5* knockout and intestinal epithelial-specific *Zdhhc5*-knockout mouse models for AOM/DSS-induced CRC. (H–J) Representative macroscopic morphologies (H), tumor numbers (I), and tumor volume (J) in WT and KO mice. (K–M) Representative macroscopic morphologies (K), tumor numbers (L), and tumor volume (M) in Ctrl and CKO mice. (N) Untargeted lipidomics of colon tissues shows accumulation of PUFA-containing lipids in CKO mice. Data are presented as mean ± SD; statistical significance was determined by Student’s *t* test; ∗*p* < 0.05, ∗∗*p* < 0.01, ∗∗∗*p* < 0.001.

Having mechanistically linked ZDHHC5-mediated β-catenin C466 palmitoylation to the transactivation of *SLC7A11*and *PD-L1*, we next assessed the translational relevance of this axis and validated its pathogenic function across physiological models. We first examined its clinical association. *ZDHHC5* mRNA was overexpressed in CRC tissues, with the highest levels in advanced-stage (III/IV) tumors (Figures S9A and S9B). TMA analysis and western blot experiments confirmed the overexpression of ZDHHC5 protein in CRC (Figures 6A, S9C, and S9D), which correlated with increased tumor volume (Figure 6B). High *ZDHHC5* expression predicted poorer overall survival (OS) and recurrence-free and disease-free survival in multiple CRC cohorts (Figures S9E–S9J). Notably, in a cohort of patients treated with anti-PD-L1 immunotherapy, high *ZDHHC5* expression remained associated with worse OS (Figure S9K). This clinical observation supports our mechanistic hypothesis that ZDHHC5 drives a broad immunosuppressive program, extending beyond PD-L1 upregulation to include the suppression of immunogenic ferroptosis.

To test this functionally, we knocked down *ZDHHC5* in CRC cells. This suppressed proliferation and induced ferroptosis hallmarks—increased lipid ROS, 4-HNE accumulation, and HMGB1 release—phenotypes rescued by SLC7A11 overexpression (Figures S10A–S10E), confirming SLC7A11 as a key effector. In patient-derived organoids, *ZDHHC5* knockdown reduced organoid size and increased extracellular HMGB1 (Figures 6C and 6D). Similarly, in HCT116-derived xenografts, *ZDHHC5* knockdown inhibited tumor growth and promoted immunogenic ferroptosis markers, with these effects reversed by SLC7A11 overexpression (Figures S10F–S10J).

We then asked if the therapeutic effect of *ZDHHC5* loss depends on SLC7A11. In patient-derived xenografts (PDXs) (Figure 6E), *ZDHHC5* knockdown significantly suppressed tumor growth and elevated plasma HMGB1 levels. Importantly, this effect was specific to PDXs exhibiting high intrinsic SLC7A11 expression and was absent in models with low expression of it (Figures 6F and S10K–S10N). This demonstrates that SLC7A11 activity is required for the anti-tumor effect of ZDHHC5 ablation.

To evaluate the pathophysiological impact of *ZDHHC*5 loss in an immunocompetent, disease-relevant context, we used the AOM/DSS-induced colitis-associated CRC model. Both intestinal epithelium-specific conditional knockout (CKO) (*Zdhhc5*^*flox/flox*^; *Villin*^*Cre+*^) and whole-body knockout mice developed significantly fewer and smaller tumors (Figures 6G–6M and S11A–S11C). Untargeted lipidomics revealed accumulation of pro-ferroptotic polyunsaturated fatty acid （PUFA） phospholipids^40^ in the colon tissues of CKO mice (Figure 6N), and tumors exhibited elevated lipid ROS and 4-HNE levels, along with decreased *SLC7A11* (Figures S11D and S11E). Strikingly, *Zdhhc5* ablation profoundly remodeled the tumor immune microenvironment, reducing *PD-L1* expression while increasing CD8^+^ T cell infiltration and plasma HMGB1 (Figures S11E–S11G).

Collectively, genetic loss-of-function studies across models demonstrate that the ZDHHC5-β-catenin axis is a critical driver of CRC. It functions by simultaneously crippling the initiation phase of anti-tumor immunity through suppression of the DAMP signal HMGB1, thereby impairing antigen presentation, and directly inhibiting the effector phase via PD-L1-mediated suppression of cytotoxic T cells.

### β-cat-oxazole, a first-in-class inhibitor of the ZDHHC5-β-catenin interaction, suppresses colorectal tumorigenesis

**Figure 7:**
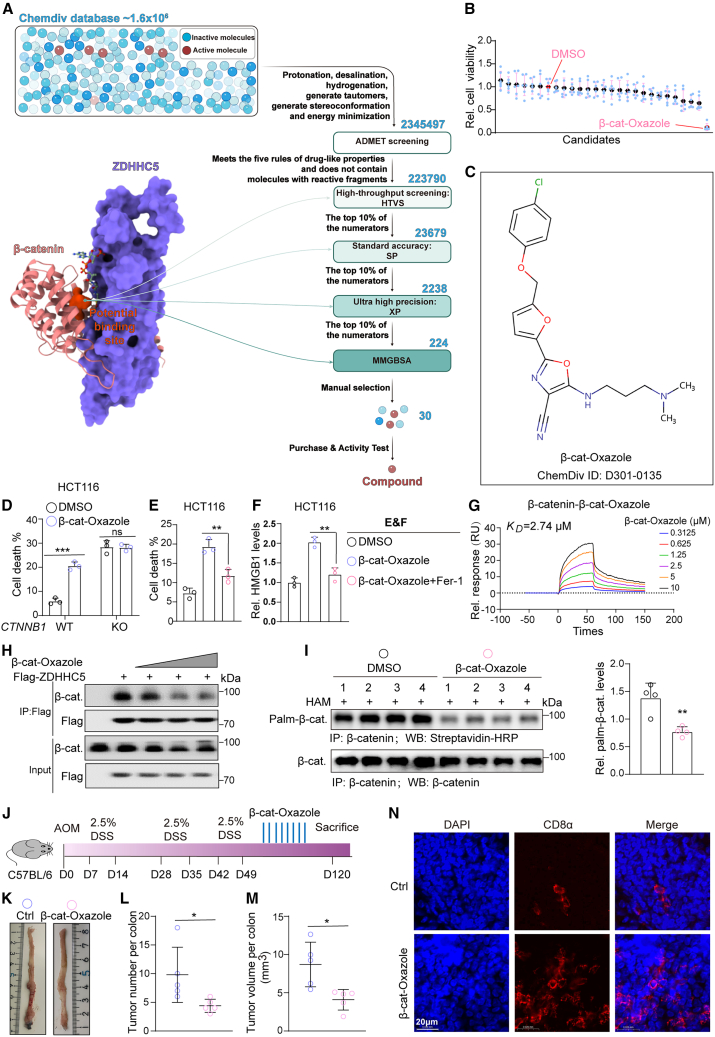
β-cat-oxazole, a first-in-class inhibitor of the ZDHHC5-β-catenin interaction, suppresses colorectal tumorigenesis (A) Virtual screening process to identify compounds targeting the ZDHHC5/β-catenin complex. (B) Cell viability of HCT116 cells after treatment with virtual screening candidates in combination with RSL3 (0.05 μM) for 5 days. Large dots represent the mean. (C) Chemical structure of β-cat-oxazole. (D) β-cat-oxazole induces cell death in a β-catenin-dependent manner. WT or *CTNNB1*-knockout HCT116 cells were treated with 10 μM β-cat-oxazole for 2 days, followed by trypan blue staining. (E and F) Ferrostatin-1 rescues β-cat-oxazole-induced cell death and HMGB1 release. HCT116 cells were treated with 5 μM β-cat-oxazole and 10 μM ferrostatin-1 for 3 days, followed by trypan blue staining (E) and measurement of HMGB1 levels in culture supernatants by ELISA (F). (G) SPR analysis of β-cat-oxazole binding to β-catenin. (H) β-cat-oxazole dose dependently disrupts the ZDHHC5-β-catenin interaction. HEK293T cells transfected with FLAG-ZDHHC5 were treated with varying concentrations of β-cat-oxazole (0, 5, 10, and 20 μM) for 2 days, followed by coIP analysis. (I) β-cat-oxazole inhibits β-catenin palmitoylation. HEK293T cells were treated with 10 μM β-cat-oxazole for 2 days, followed by ABE assay (left). Quantification of β-catenin palmitoylation from the left panel (right). (J) Schematic of the AOM/DSS-induced CRC mouse model. Mice were treated with 20 mg/kg β-cat-oxazole intraperitoneally every 3 days for 8 doses. (K–M) Representative macroscopic morphologies (K), tumor numbers (L), and tumor volume (M) in DMSO- or β-cat-oxazole-treated mice. (N) β-cat-oxazole treatment promotes CD8^+^ T cell infiltration in mouse CRC. AOM/DSS-induced CRC tumors in mice treated with β-cat-oxazole were subjected to immunofluorescence analysis. Data are presented as mean ± SD; statistical significance was determined by Student’s *t* test; ∗*p* < 0.05, ∗∗*p* < 0.01, ∗∗∗*p* < 0.001.

Having established the ZDHHC5-β-catenin axis as a critical driver of CRC, we next explored its therapeutic druggability. In the absence of specific ZDHHC5 inhibitors, we aimed to directly inhibit the pathogenic palmitoylation of β-catenin at C466 by disrupting the ZDHHC5-β-catenin interaction. We performed a structure-based virtual screen of 1.6 million compounds, targeting the predicted ZDHHC5-β-catenin protein-protein interaction interface surrounding C466 (Figure 7A). From the top 30 ranked candidates, 26 commercially available analogs were procured for biological validation. Among these, β-cat-oxazole (named for its structure and novelty) most potently enhanced HCT116 cell sensitivity to a ferroptosis inducer (Figures 7B and 7C).

Biological characterization confirmed that β-cat-oxazole induced cell death in a β-catenin-dependent manner (Figure 7D), and this death was rescued by the ferroptosis inhibitor ferrostatin-1 (Figure 7E). Treatment also triggered the extracellular release of HMGB1, which was similarly blocked by ferrostatin-1 (Figure 7F), confirming the immunogenic nature of the cell death. Molecular dynamics simulations and surface plasmon resonance (SPR) analysis revealed that β-cat-oxazole stably binds to β-catenin with high affinity (K_D_ = 2.74 μM), forming critical interactions with residues including Arg469, Lys508, Arg515, Glu568, and Glu571 (Figures 7G and S12A–S12I). Importantly, mutation of these key β-catenin residues abolished the ability of β-cat-oxazole to induce its pro-ferroptotic effects, including cell death and the elevation of 4-HNE, lipid ROS, and extracellular HMGB1 (Figures S12J–S12M). Functionally, β-cat-oxazole dose-dependently disrupted the ZDHHC5-β-catenin interaction and reduced cellular β-catenin palmitoylation levels (Figures 7H and 7I).

A comprehensive safety assessment showed that β-cat-oxazole selectively reduced the viability of CRC cell lines but not the normal colon epithelial cell line NCM460 *in vitro* (Figures S13A and S13B). In a 28-day repeated-dose study *in vivo*, β-cat-oxazole (20 mg/kg) was well tolerated, with no significant changes in body weight, serum biochemistry, or histopathology of major organs (Figures S13C–S13F).

We then evaluated the therapeutic efficacy of β-cat-oxazole *in vivo*. Initial assessment in a subcutaneous MC38 tumor model in immunocompetent mice demonstrated that β-cat-oxazole treatment effectively inhibited tumor growth (Figures S14A and S14B). Mechanistically, tumors from treated mice exhibited on-target pathway modulation, including downregulation of *SLC7A11* and *PD-L1*, elevated markers of ferroptosis (lipid ROS, 4-HNE) and immunogenicity (HMGB1) (Figures S14C–S14G). We further validated its efficacy in a more disease-relevant, immunocompetent AOM/DSS-induced colitis-associated CRC model. β-cat-oxazole treatment initiated after tumor establishment significantly reduced tumor burden (Figures 7J–7M). Consistently, tumors from treated mice recapitulated the same pattern of concurrent *SLC7A11* and *PD-L1* downregulation, elevated ferroptosis (4-HNE) and immunogenic marker (HMGB1), and increased CD8^+^ T cell infiltration (Figures 7N and S14H–S14K).

Collectively, these data identify β-cat-oxazole as a first-in-class, proof-of-concept inhibitor that directly targets the β-catenin palmitoyl-switch. By disrupting the ZDHHC5-β-catenin interaction, β-cat-oxazole phenocopies the genetic ablation of this axis, synchronously reactivating immunogenic ferroptosis and ablating PD-L1-mediated immune evasion to suppress CRC progression.

## Discussion

This study identifies ZDHHC5 as the palmitoyltransferase for β-catenin at C466 in CRC and delineates the coordinated immunosuppressive program governed by this modification. We demonstrate that ZDHHC5-catalyzed palmitoylation of β-catenin at C466 establishes a central regulatory node, integrating the major resistance pathways of immunogenic ferroptosis and immune evasion.

Our findings reveal a competitive PTM crosstalk at β-catenin C466, where palmitoylation acts as a deterministic molecular switch by displacing inhibitory S-nitrosylation. This enhances the formation and transcriptional activity of the β-catenin/TCF4 complex. This work shifts the paradigm from viewing PTMs as isolated events to understanding their dynamic competition as a binary switch that reprograms β-catenin from a canonical proliferative driver to an immunomodulatory transcription factor.

Critically, this palmitoyl-switch steers β-catenin toward a transcriptional program, coordinately upregulating *SLC7A11* to suppress immunogenic ferroptosis and *PD-L1* to inhibit cytotoxic T cells. Our *in vivo* epistasis experiment confirms these two arms are functionally integrated; HMGB1 release is required to unlock the efficacy of PD-L1 blockade. Thus, the ZDHHC5-β-catenin axis orchestrates a dual-hit immunosuppressive strategy, crippling both the initiation and effector phases of the cancer-immunity cycle.

Our delineation of the pathogenic role of the ZDHHC5-β-catenin axis prompts consideration of its physiological functions. ZDHHC5 is broadly expressed and regulates fundamental processes such as synaptic plasticity.^41^^,^^42^^,^^43^^,^^44^ The absence of severe developmental defects in our *Zdhhc5*-knockout models likely reflects functional redundancy within the ZDHHC family under physiological conditions. In stark contrast, within the pathological, hyperactive Wnt/β-catenin signaling environment of CRC, the ZDHHC5-mediated palmitoylation of β-catenin at C466 becomes a critical, non-redundant dependency for immune evasion. This divergence between physiological redundancy and pathological essentiality underscores a potentially wide therapeutic window for targeted intervention.

The evolutionary conservation of this regulatory switch implies a fundamental homeostatic function, likely engaged during tissue repair and resolution of inflammation to transiently establish a protective niche. Colorectal tumor cells appear to have hijacked and constitutively activated this transient program, repurposing it for sustained immune evasion.

The development of β-cat-oxazole, a first-in-class inhibitor that disrupts ZDHHC5-β-catenin interaction, provides proof-of-concept for targeting PTM-governing protein interfaces. Unlike broad catalytic inhibitors,^45^^,^^46^ this substrate-selective strategy may minimize off-target effects. Clinically, high *ZDHHC5* expression predicts poor survival and resistance to anti-PD-L1 therapy. Our PDX models further indicate that tumors with high SLC7A11—indicative of active palmitoyl-switch signaling—are most vulnerable to inhibition of this axis, providing a mechanistic basis for patient stratification.

In summary, by elucidating a druggable β-catenin palmitoyl switch that coordinately suppresses immunogenic signaling from ferroptosis (notably HMGB1 release) while modulating the adaptive immune checkpoint PD-L1, our findings nominate PTM interface targeting as a transformative strategy in precision oncology.

### Limitations of the study

While our loss-of-function studies and the specific phenotype induced by β-cat-oxazole robustly establish β-catenin C466 palmitoylation as the predominant pathway mediating the described phenotypes, several limitations point to future directions. First, although our functional data strongly support the ZDHHC5-β-catenin interaction, ZDHHC5 is an integral membrane protein. Our computational modeling, while predictive, does not fully account for the potential influence of the native membrane lipid environment on the interaction geometry. Second, a comprehensive understanding of the upstream regulatory network controlling ZDHHC5 activity and its full substrate repertoire in CRC remains an important area for future investigation. Third, definitive pharmacokinetic and pharmacodynamic profiling of β-cat-oxazole will be crucial for its translational development. Fourth, while our cohorts included both sexes, the study was not designed to analyze the influence of sex (biological attribute) or gender (sociocultural construct) on the palmitoyl-switch pathway and its role in immune evasion. Future dedicated studies are needed to address this. Finally, future studies should also explore the generality of this mechanism in other β-catenin-driven malignancies and evaluate combination therapies with PD-1/PD-L1 inhibitors.

## Resource availability

### Lead contact

Additional information and requests for resources and reagents should be addressed to the lead contact, Ping Lan (lanping@mail.sysu.edu.cn).

### Materials availability

This study did not generate new unique materials or reagents.

### Data and code availability

• The RNA-seq data generated in this study are publicly available in the NCBI Sequence Read Archive (SRA) under BioProject accession number PRJNA1348236. The transcriptomic and clinical data used in this study are publicly available in the following resources: the CRC cases on the TIMER website (https://compbio.cn/timer1/), TNMplot (https://tnmplot.com/analysis/), and the GENT website (http://gent2.appex.kr/gent2/). Additional data can be accessed from the GEO database under the accession numbers GSE17537, GSE29623, and GSE132465. Public spatial transcriptomics data are available in the study by Wu et al.^38^
• This paper does not report original code.
• Any additional information required to reanalyze the data reported in this paper is available from the lead contact upon request.

## Acknowledgments

This work was supported by grants from the 10.13039/501100001809National Natural Science Foundation of China (82573793), 10.13039/501100004791Shenzhen Medical Research Found (A2503028), Shenzhen Science and Technology Program (JCYJ20240813151114019 and szbo202313), and Guangdong Basic and Applied Basic Research (2024A1515013221).

## Author contributions

Q.Z. initiated the entire study. P.L., Z.H., G.T., and Q.Z. supervised the project. Q.Z. designed and performed all the experiments and related analysis and wrote the manuscript. Y.K. and Y.L. performed most experiments and related analysis. X.L., L.W., Z.L., X.Y., Y.X., D.L., W.Y., X.C., and Y.S. designed and performed plasmid construction and most animal experiments and collected and analyzed data. Q.Y. performed protein structure analysis, virtual screening, and molecular dynamics analysis. K.C. designed and performed scRNA-seq and spatial transcriptomics analyses and contributed to the completion of the summary figure. J.Y. provided CRC TMA and technical help with animal experiments. All authors discussed the results.

## Declaration of interests

The authors declare no competing interests.

## STAR★Methods

### Key resources table

**Table:** 

REAGENT or RESOURCE	SOURCE	IDENTIFIER
**Antibodies**		

Rabbit polyclonal anti-SLC7A11	Proteintech	Cat# 26864-1-AP; RRID: AB_2880661
Rabbit polyclonal anti-ZDHHC5	Santa Cruz	Cat# 21324-1-AP; RRID: AB_10732816
Rabbit monoclonal anti-β-catenin	Abcam	Cat# Ab32572; RRID: AB_725966
Mouse monoclonal anti-β-actin	Affinity	Cat# T0022; RRID: AB_2839417
Mouse monoclonal anti-β-actin	Zenbio	Cat# 200068-8F10; RRID: AB_2722710
Rabbit monoclonal anti-HA	Sigma	Cat# H6908; RRID: AB_260070
Mouse monoclonal anti-HA	Abclonal	Cat# AE008; RRID: AB_2770404
Rabbit polyclonal anti-Flag	Proteintech	Cat# 20543-1-AP; RRID: AB_11232216
Rabbit monoclonal anti-β-catenin	Abclonal	Cat# A19657;RRID: AB_2862719
Rabbit monoclonal anti-β-catenin	CST	Cat# 8480; RRID: AB_11127855
*InVivo*MAb anti-mouse PD-L1 (B7-H1)	BioXcell	Cat# BE0101; RRID: AB_10949073
HRP-conjugated Streptavidin	Sangon Biotech	Cat# D111054

**Bacterial strains**		

*Escherichia coli BL21*	Thermo	Cat# EC0114

**Biological samples**		

Human CRC sample	This study	N/A

**Chemicals, peptides, and recombinant proteins**		

Ferrostatin-1	MCE	Cat# HY-100579
iCRT14	Santa Cruz	Cat# sc-362746
N-Ethylmaleimide	Sangon Biotech	Cat# A600450-0005
BMCC-biotin [(1-Biotinamido)-4-[4’-(maleimidomethyl)cyclohexanecarboxamido]hexane]	Sangon Biotech	Cat# C100222-0050
Anti-Flag agarose beads	Selleck	Cat# B23101
Neocuproine	Sangon Biotech	Cat# A600651-0001
Acetone	Sangon Biotech	Cat# A375081-0005
Biotin-HPDP (N-[6-(biotinamido)hexyl]-3’-(2′-pyridyldithio)propionamide)	Sangon Biotech	Cat# A425727-0001
Sodium ascorbate	Sangon Biotech	Cat# A500830-0100
Anti-HA agarose beads	Shenzhen KangTi Life Technology Co. Ltd	Cat# KTSM1305
DMEM medium	Gibco	Cat# C11995500BT
Fetal bovine serum	Gibco	Cat# 10099141C
Penicillin-streptomycin	Gibco	Cat# 15140122
S-methylmethanethiosulfonate	MACKLIN	Cat# S831262-1g
Paraformaldehyde	Aladdin	Cat# C104190
RSL3	MCE	Cat# HY-100218A
Necrostatin-1	MCE	Cat# HY-15760
3-Methyladenine	MCE	Cat# HY-19312
Z-VAD-FMK	MCE	Cat# HY-16658B
Purified β-catenin protein	MCE	Cat# HY-P74382
β-cat-Oxazole	ChemDiv	Cat# D301-0135
HIT103018504	ChemDiv	Cat# 4296-0131
HIT107432567	ChemDiv	Cat# 3998-0008
HIT100485268	ChemDiv	Cat# 8017-4194
HIT101585604	ChemDiv	Cat# 8017-7834
HIT214960127	ChemDiv	Cat# 8015-3437
HIT100057845	ChemDiv	Cat# D126-0068
HIT103652498	ChemDiv	Cat# 5826-0308
HIT105526676	ChemDiv	Cat# 8011-9092
HIT101747327	ChemDiv	Cat# D715-1232
HIT100127485	ChemDiv	Cat# Y021-4543
HIT211464510	ChemDiv	Cat# E797-0226
HIT107522630	ChemDiv	Cat# 3448-4268
HIT102508788	ChemDiv	Cat# 8020-1175
HIT102575499	ChemDiv	Cat# Y041-2644
HIT106369330	ChemDiv	Cat# 3998-0021
HIT102147003	ChemDiv	Cat# Y042-8163
HIT100873016	ChemDiv	Cat# C784-3271
HIT100784760	ChemDiv	Cat# Y041-2645
HIT100104674	ChemDiv	Cat# D715-1142
HIT102003142	ChemDiv	Cat# 8008-6912
HIT104067946	ChemDiv	Cat# G415-3000
HIT102563148	ChemDiv	Cat# 8016-1084
HIT104914904	ChemDiv	Cat# Y020-3349
HIT105537516	ChemDiv	Cat# 8012-6803
HIT214960057	ChemDiv	Cat# 8012-7041
BODIPY C11 probe	Thermo	Cat# D3861
DAPI	Sigma-Aldrich	Cat# D9542

**Critical commercial assays**		

Cell counting kit 8	Glpbio	Cat# GK10001
SYBR Green Real-time PCR Master Mix	Biomarker	Cat# RM02001
Dual-luciferase reporter assays kit	Vazyme	Cat# DL-101-01
Immunofluorescence reagent kit	PANOVUE	Cat# 0001100020
4-HNE ELISA kit	Jiangsu Meimian industrial Co., Ltd	Cat# MM-925888O1
Human HMGB1 ELISA kit	Jiangsu Meimian industrial Co., Ltd	Cat# MM-13713H1
Mouse Hmgb1 ELISA kit	Jiangsu Meimian industrial Co., Ltd	Cat# MM-44107M1
ROS ELISA kit	Jiangsu AIDISHENG Biotechnology Co., Ltd	Cat# ADS-W-FM016
ALT assay kit	Jiangsu AIDISHENG Biotechnology Co., Ltd	Cat# ADS-W-AJS001-96
AST assay kit	Jiangsu AIDISHENG Biotechnology Co., Ltd	Cat# ADS-W-AJS002-96
UREA assay kit	Jiangsu AIDISHENG Biotechnology Co., Ltd	Cat# ADS-W-N013-96
CREA assay kit	Jiangsu AIDISHENG Biotechnology Co., Ltd	Cat# ADS-W-FM034
Hiscript@ III RT Super Mix with gDNA wiper	Vazyme	Cat# R323-01
Total RNA isolation kit	Vazyme	Cat# RC112-01

**Deposited data**		

TIMER	N/A	https://compbio.cn/timer1/
TNMplot	N/A	https://tnmplot.com/analysis/
GENT	N/A	http://gent2.appex.kr/gent2/
RNA-seq data	SRA	PRJNA1348236
GSE17537	GEO	https://www.ncbi.nlm.nih.gov/geo/query/acc.cgi?acc=GSE17537
GSE29623	GEO	https://www.ncbi.nlm.nih.gov/geo/query/acc.cgi?acc=GSE29623
GSE132465	GEO	https://www.ncbi.nlm.nih.gov/geo/query/acc.cgi?acc=GSE132465

**Experimental models: Cell lines**		

HCT116	ATCC	Cat# CCL-247; RRID: CVCL_0291
DLD1	ATCC	Cat# CCL-221; RRID: CVCL_0248
HEK293T	ATCC	Cat# CRL-3216; RRID: CVCL_0063

**Experimental models: Organisms/strains**		

BALB/c-Nude mice	Guangzhou Ruige Biological Technology Co., Ltd	N/A
*Zdhhc5*^*flox/flox*^ Mice	Cyagen	N/A
*Villin*^*Cre*^ mice	Cyagen	N/A

**Oligonucleotides**		

Primers for constructs are listed in Table S4	This study	N/A
Primers for qPCR are listed in Table S4	This study	N/A
shRNA or sgRNA sequences are listed in Table S4	This study	N/A

**Recombinant DNA**		

shCtrl	This study	N/A
shZDHHC5-1#	This study	N/A
shZDHHC5-2#	This study	N/A
shSLC7A11	MIAOLING PLASMID	Cat# P59373
shβ-catenin	This study	N/A
shHmgb1	MIAOLING PLASMID	Cat# P58689
sgCtrl	This study	N/A
sgCTNNB1-1#	This study	N/A
sgCTNNB1-2#	This study	N/A
pCMV-HA vector	This study	N/A
pGL3-basic luciferase vector	This study	N/A
LentiCRISPRv2	This study	N/A
pLKO.1-puro vector	This study	N/A
pHAGE-CMV-MCS-PGK vector	This study	N/A
Flag-β-catenin	This study	N/A
HA-β-catenin	This study	N/A
β-catenin C466A	This study	N/A
Flag-ZDHHC5	This study	N/A
HA-ZDHHC5	This study	N/A
ZDHHC5 C314S	This study	N/A
Flag-TCF4	MIAOLING PLASMID	Cat# P69042
Flag-SLC7A11	MIAOLING PLASMID	Cat# P77538
β-catenin mut	Tsingke	N/A
β-catenin MUT5A	Tsingke	N/A
ZDHHC5 mut	Tsingke	N/A
PGL3-SLC7A11-promoter	This study	N/A
TBE1-MUT	This study	N/A
TBE2-MUT	This study	N/A
Primer for construction	This study	Table S4

**Software and algorithms**		

ImageJ software	NIH	https://imagej.nih.gov/ij/
Adobe illustrator 2021	Adobe	https://www.adobe.com/
Graphpad Prism 9 software	GraphPad	https://www.graphpad.com/scientific-software/prism/
GROMACS package (version 2023.03	N/A	https://www.sciencedirect.com/science/article/pii/S2352711015000059
TIP3P water model	N/A	https://www.scopus.com/pages/publications/0004016501
GSEA software	N/A	https://www.gsea-msigdb.org/gsea/index.jsp
FASTQC software (v0.11.9)	N/A	https://www.bioinformatics.babraham.ac.uk/projects/fastqc/
HISAT2 (v2.2.1)	N/A	https://daehwankimlab.github.io/hisat2/
R studio software	RStudio	https://www.rstudio.com/

### Experimental model and study participant details

#### Human subjects

Ten pairs of tumor and adjacent normal tissues were consecutively selected from our biobank based on sample availability and RNA integrity, and allocated as a single cohort for palmitoylation level comparison between tumor and normal tissues. A tissue microarray was constructed from 237 pairs of tumor and adjacent normal tissues. This cohort was used as a wholeour assess protein expression correlations and clinical associations. Five fresh CRC sample pairs were selected based on tissue availability and viability for implantation. They were first analyzed by Western blot for SLC7A11 expression. Based on the results, they were allocated into two experimental groups: a high-SLC7A11 expression group and a low-SLC7A11 expression group, for subsequent PDX model establishment and *in vivo* studies. No statistical method was used to pre-determine sample size for the discovery cohorts (*N* = 10, *N* = 237). The sample size for the PDX cohort (*N* = 5) was determined by resource availability and feasibility for the *in vivo* proof-of-concept experiment. All samples were obtained from the Sixth Affiliated Hospital of Sun Yat-sen University. Detailed clinical characteristics for each cohort are provided in Tables S1, S2, and S3.

#### Animal studies

*Zdhhc5*^*flox/flox*^ and *Villin*^*Cre*^ mice were purchased from Cyagen (Suzhou, China). All animal experiments were approved by the Animal Care Committee of Guangzhou Ruige Biological Technology Co., Ltd. Mice were housed in groups of 4–5 in specific pathogen-free (SPF) conditions within microisolator cages at the Experimental Animal Center of Guangzhou Ruige Biological Technology Co., Ltd.

*Zdhhc5*^*flox/flox*^ mice were crossed with *Villin*^*Cre*^ mice to generate intestine-specific knockout mice (*Zdhhc5* CKO; *Zdhhc5*^*flox/flox*^*;Villin*^*Cre+*^), with their *Zdhhc5*^*flox/flox*^;*Villin*^*Cre -*^littermates serving as WT controls. For the global *Zdhhc5* knockout model, sustained breeding of the colony was achieved by crossing *Zdhhc5*^*+/−*^ mice. This strategy was employed because global homozygous knockout results in male infertility,^47^ preventing the establishment of a breeding pair between homozygous individuals. Mendelian inheritance was observed in the offspring from heterozygous crosses, yielding *Zdhhc5*^−/−^, *Zdhhc5*^*+/−*^, and WT littermates for experiments. Genotyping was performed by PCR analysis of tail DNA using primers specific for the Zdhhc5 floxed and Villin-Cre alleles.

For the subcutaneous xenograft model, six-week-old female BALB/c nude mice were injected subcutaneously with 5×10^6^ HCT116 cells resuspended in 100 μL of PBS. Tumors were harvested 30 days post-injection for subsequent experiments. Additionally, 1 × 10^6^ MC38 cells resuspended in 100 μL PBS were injected subcutaneously into C57BL/6J WT mice. For the anti-PD-L1 treatment, tumors received 100 μg of anti-PD-L1 on days 14 and 18. For the β-cat-Oxazole treatment, mice were intraperitoneally injected with 20 mg/kg of β-cat-Oxazole every 3 days for a total of 5 doses. Tumor volumes were measured using the formula: V (mm^3^) = a×b^2^/2, where a represents length and b represents width.

For PDX models, tumor tissues from CRC patients were transplanted subcutaneously into six-week-old female BALB/c nude mice. Upon tumor growth, the subcutaneous tumors were excised, dissociated into fragments of approximately 1 mm, and re-implanted into new BALB/c nude mice. One week post-transplantation, mice bearing tumors of comparable size (ranging from 50 to 100 mm^3^) were randomly assigned to two groups. To sustain lentivirus-mediated gene knockdown throughout the experiment, mice received intratumoral injections, administered at multiple sites to enhance distribution, of lentivirus expressing either a control or shZDHHC5 every other day for a total of 10 injections. This multi-injection protocol was employed to counteract the transient nature of *in vivo* lentiviral transduction and ensure persistent target suppression. Tumor volumes and immunohistochemical analysis were performed at day 28. Tumor volumes were calculated using the same formula as described above.

For the AOM/DSS-induced CRC model, eight-week-old C57BL/6 mice were intraperitoneally injected with 10 mg/kg AOM (Sigma-Aldrich). After 7 days, mice were given drinking water containing 2.5% DSS (MP Biomedicals, Santa Ana, CA, USA) for one week, followed by regular drinking water for 2 weeks. This cycle was repeated with 2.5% DSS for another week, and mice were sacrificed on day 120. For β-cat-Oxazole treatment, after the third round of DSS administration, mice were intraperitoneally injected with 20 mg/kg β-cat-Oxazole every 3 days for a total of 8 doses. Mice were then sacrificed, and colon tumor tissues were harvested for subsequent analysis.

#### Cell culture

The human cell lines (HCT116, DLD1, HEK293T) were obtained from the American Type Culture Collection (ATCC) and authenticated by STR profiling. All cells tested negative for mycoplasma contamination. These cells were grown in DMEM medium (Gibco, NY, USA) supplemented with 10% fetal bovine serum (Gibco, NY, USA) and 1% penicillin-streptomycin (Gibco, CA, USA), maintained at 37°C in a 5% CO2 incubator.

#### Organoid culture

Organoids were cultured following a previously established protocol.^48^ Briefly, human intestinal tumor epithelial cells were maintained in advanced DMEM/F12 medium supplemented with growth factors, including 100 ng/mL Noggin (Peprotech), 500 ng/mL R-spondin (Peprotech), 50 ng/mL epidermal growth factor (Peprotech), and 10 μM Y-27632 (Abmole). PDOs were subsequently infected with lentivirus expressing shZDHHC5. After infection, PDOs were photographed, and their diameters were measured.

### Method details

#### MS analysis

HEK293T cells transfected with Flag-β-catenin or Flag-ZDHHC5 plasmids were lysed and immunoprecipitated using anti-Flag agarose. Following SDS-PAGE and Coomassie Blue staining, the protein bands were excised, subjected to in-gel trypsin digestion, and dried. Protein composition was then analyzed by MS as previously described.^49^

#### Stable cell lines

Lentiviral production and transduction were performed as previously described.^50^ Briefly, lentiviral vectors containing the target constructs were packaged in HEK293T cells. Tumor cells were then infected with the lentiviruses in the presence of polybrene. Infected cells were selected with 1 μg/mL puromycin for two weeks to establish stable clones. The expression of the target proteins in these clones was confirmed by Western blot analysis.

#### Co-immunoprecipitation and immunoblot analysis

Co-immunoprecipitation and immunoblot analyses were performed as previously described.^50^ Briefly, cells transfected with the desired plasmids were lysed in 1 mL of lysis buffer. For co-immunoprecipitation, anti-Flag or anti-HA agarose beads were washed three times with 1 mL lysis buffer before being incubated with 1 mL of cell lysate overnight at 4°C. The following day, the beads were centrifuged, and the supernatant was discarded. The beads were washed three times, resuspended in 2× SDS sample buffer, and boiled for 10 min. The resulting samples were analyzed by immunoblotting using the appropriate antibodies.

#### Real-time quantitative PCR (RT-qPCR)

RT-qPCR was performed as previously described.^10^ Briefly, total RNA was extracted from cells or tissues, followed by reverse transcription to generate complementary DNA (cDNA). The cDNA was then subjected to qPCR using SYBR Green Supermix (Bio-Rad, Hercules, CA) and standard protocols. Primer sequences for qPCR are provided in Table S4. GAPDH was used as an internal control to normalize the data.

#### Promoter analysis

The promoter sequence of the human *SLC7A11* gene was obtained from the UCSC Genome Browser. Specifically, we focused on approximately 2000 bp upstream of the ATG coding region. Using bioinformatics tools, we performed a sequence alignment of the retrieved SLC7A11 promoter with known β-catenin/TCF4 binding motifs documented in the literature. The consensus sequence for the β-catenin/TCF4 complex is described as (-CTTTGA/TA/T-).^48^

#### Chromatin immunoprecipitation assay

The chromatin immunoprecipitation assay was performed according to a previously established protocol.^10^ Briefly, cells were cross-linked *in situ* with 1% formaldehyde for 10 min, followed by quenching with 0.125 M glycine for 5 min at room temperature. Cells were then lysed in SDS lysis buffer, and chromatin DNA was fragmented by sonication to a size range of 200–1000 base pairs. The supernatant was diluted 10-fold in ChIP dilution buffer and precleared with 50 μL agarose beads for 2 h at 4°C. After centrifugation to collect the supernatant, specific antibodies (3 μg) were added, and the mixture was rotated overnight at 4°C. The following day, 50 μL of agarose beads were added and incubated for an additional 2 h at 4°C. Finally, the de-crosslinked DNA was analyzed by qPCR using specific primers listed in Table S4.

#### Luciferase reporter assay

The luciferase reporter assay was performed as previously described.^10^ Briefly, 0.3 μg of the pGL3 vector expressing TREs or the indicated mutant, along with 50 ng of Renilla luciferase reporter, were transfected in triplicates into HEK293T cells. After 36 h, luciferase activity was measured using the Dual-Luciferase Reporter Assay System. Renilla activity was used as the internal control for normalization.

#### Immunohistochemical (IHC) analysis

CRC samples were fixed and embedded in paraffin according to standard protocols. IHC analysis was performed using antibodies against the indicated protein. The staining results were evaluated based on both intensity and the proportion of tumor cells showing a positive reaction. Staining intensity was scored as follows: 0 for negative, 1 for weak, 2 for moderate, and 3 for strong. The frequency of positive cells was scored as: 0 for <5%, 1 for 5–25%, 2 for 25–50%, 3 for 50–75%, and 4 for >75%. The total score, ranging from 0 to 12, was calculated by multiplying the intensity score by the score for the percentage of positive cells.

#### Immunofluorescence analysis

Immunofluorescence analysis was performed according to the manufacturer’s protocol (PANOVUE, Beijing, China). Briefly, cells seeded on glass slides were fixed with 4% paraformaldehyde for 15 min at room temperature (RT), permeabilized with 0.5% Triton X-100 for 15 min at RT, and blocked with 5% fetal bovine serum. Primary antibodies, diluted in blocking buffer, were applied overnight at 4°C. After washing with TBST buffer, samples were incubated with HRP-conjugated secondary antibodies for 10 min at RT. Following additional TBST washes, tyramide signal amplification (TSA) was performed using fluorescent dyes (PPD570 and PPD650) as per the manufacturer’s instructions (Panorama, China). Nuclei were counterstained with DAPI, and images were acquired using a laser scanning confocal microscope.

#### Measurement of ROS and lipid ROS levels

Lipid ROS, a subset of ROS, are established hallmarks of ferroptosis.^51^ ROS levels were measured using a ROS ELISA kit purchased from AIDISHENG (Jiangsu, China), following the manufacturer’s standard protocol. Briefly, DCFH-DA probe was diluted 1:1000 in serum-free medium to a final concentration of 10 μM. After removing the cell culture medium, the diluted DCFH-DA was added to ensure sufficient coverage of the cells, with at least 1 mL per well in a 6-well plate. The cells were incubated with the hydrogen donor for 30 min at 37°C. After incubation, the medium was aspirated, and the cells were gently washed with serum-free medium until nearly all the cell layer detached. The cell suspension was collected in a 1.5 mL centrifuge tube, washed twice to remove unpenetrated probe, and centrifuged at 1000 rpm for 5 min. The supernatant was discarded, and the cells were resuspended in PBS. ROS levels were measured using a microplate reader with excitation at 488 nm and emission at 525 nm. For lipid ROS detection, CRC cells were seeded in a 6-well plate, washed twice with Hank’s balanced salt solution (Gibco), and incubated in the dark at 37°C for 30 min with 10 μmol/L BODIPY C11 probe (Thermo Fisher Scientific). After staining, cells were analyzed by flow cytometry.

#### Detection of 4-HNE

4-HNE levels were measured using a 4-HNE ELISA kit purchased from Jiangsu Meimian Industrial Co., Ltd., following the manufacturer’s standard protocol. Briefly, 1×10^6^ cells were collected, resuspended in PBS (pH 7.2–7.4), and disrupted by sonication to release intracellular contents. The cell lysates were centrifuged at 3000 rpm for 20 min at 4°C. Then, 50 μL of the supernatant was added to a 4-HNE-coated ELISA plate and incubated at room temperature for 10 min. After incubation with anti-4-HNE antibody and secondary antibody-HRP, substrate solution was added, and the absorbance at 450 nm was measured.

#### Determination of HMGB1 release in cell culture supernatants

HMGB1 concentration in cell culture supernatants was measured using a commercial ELISA kit. Briefly, supernatants were collected and centrifuged at 1000 × g for 20 min at 2°C-8°C to remove debris. The clarified supernatants were either assayed immediately or stored at −80°C. A sandwich ELISA was performed by adding 50 μL of standards or samples to the antibody-precoated microplate. After adding 100 μL of HRP-conjugated detection antibody, the plate was incubated for 60 min at 37°C. Following four washes with Wash Buffer, 50 μL of Substrate A and B were added, followed by a 15-min incubation in the dark. The reaction was stopped with 50 μL of Stop Solution, and absorbance was measured at 450 nm. HMGB1 concentration was calculated from the standard curve.

#### Measurement of plasma HMGB1 levels

Blood from experimental mice was collected into EDTA or heparinized tubes, centrifuged at 1000×g for 15 min at 4°C within 30 min of collection. Plasma was carefully harvested, aliquoted, and stored at −80°C, avoiding repeated freeze-thaw cycles. For the HCT116-derived xenograft model, plasma HMGB1 was measured using a Human HMGB1 ELISA kit; for the MC38-derived xenograft and AOM/DSS-induced CRC model, a Mouse HMGB1 ELISA kit was used. The assay was performed as follows: components of the ELISA kit were equilibrated at room temperature for 20 min. Then, 50 μL of standard solution (standard wells), plasma samples (sample wells), and sample diluent (blank wells) were added to the plate. Afterward, 100 μL of enzyme-conjugated antibody was added to each well, and the plate was incubated at 37°C for 60 min. Following incubation, the liquid was discarded, and the wells were washed five times with wash buffer and dried on absorbent paper. Substrates A and B (50 μL each) were added to each well, and the plate was incubated at 37°C in the dark for 15 min for color development. The reaction was terminated by adding 50 μL of stop solution, and absorbance at 450 nm was measured within 15 min using a microplate reader. HMGB1 concentrations were calculated by plotting the logarithm of the standard concentrations (X axis) against the corresponding OD values (Y axis) and using the resulting standard curve.

#### RNA sequence

Total RNA was isolated from 1×10^7^ DLD1, HCT116 cells, or 200 mg of mouse colon tissue using TRIzol reagent according to the manufacturer’s instructions (TransGen Biotech). RNA libraries were constructed by Majorbio (Shanghai, China) and sequenced on the Illumina NovaSeq platform. Raw RNA-seq data were assessed and quality-controlled using FASTQC software (v0.11.9). The data were then aligned to the GRCm39 or GRCh38 genome using HISAT2 (v2.2.1) and gene counts were calculated with FeatureCounts (v1.28.1).

#### Untargeted lipidomics

Untargeted lipidomics was performed by METWARE (Wuhan, China) using eight colon tissue samples from three WT and five CKO mice. Briefly, samples were homogenized with a ball mill (MM400, Retsch) and analyzed on an ExionLC AD UPLC–QTRAP 6500+ mass spectrometry system (SCIEX) coupled with a Thermo Accucore C30 column. Gradient elution was performed using acetonitrile/water and acetonitrile/isopropanol mixtures containing 0.1% formic acid and 10 mM ammonium formate. Mass spectrometry was conducted with an electrospray ionization (ESI) source at 500°C; the ion spray voltage was 5500 V in positive mode and −4500 V in negative mode. Source gases were set as follows: GS1 45 psi, GS2 55 psi, curtain gas (CUR) 35 psi; collision-activated dissociation (CAD) was set to Medium. In the triple quadrupole, each ion pair was monitored using optimized declustering potential (DP) and collision energy (CE). Lipid identification was based on a custom MWDB database, and quantification was performed in multiple reaction monitoring (MRM) mode using the triple quadrupole mass spectrometer.

#### Palmitoylation assays

Protein palmitoylation was detected using the ABE method.^52^ Briefly, cells transfected with the indicated plasmids were lysed in 1 mL lysis buffer containing 50 mM NEM, followed by centrifugation (20 min, 12,000 rpm, 4°C) and overnight immunoprecipitation with anti-Flag or β-catenin-specific agarose beads. After three washes, the precipitates were divided into two equal portions: one for the –HAM control and the other for the +HAM treatment, which was incubated for 1 h at room temperature. The precipitates were then washed once with wash buffer (1 M Tris-HCl, pH 6.5) and incubated with BMCC-biotin buffer (50 mM Tris-HCl, pH 6.5, 150 mM NaCl, 5 mM EDTA, 1% Triton X-100, and 5 μM BMCC-biotin) for 1 h at 4°C. The samples were washed twice with wash buffer, followed by SDS-PAGE and blotting. Palmitoylated β-catenin was detected using HRP-conjugated streptavidin (Sangon Biotech; 1:200 in 0.5% BSA).

#### S-nitrosylation assay

S-Nitrosylation was detected using the biotin switch assay.^13^^,^^53^ Briefly, lysates from transfected HCT116 or HEK293T cells were incubated in HENS buffer (250 mM HEPES, 1 mM EDTA, 0.1 mM Neocuproine, 2% SDS, pH 7.7) with 20 mM S-methylmethanethiosulfonate (MMTS) at 50°C for 20 min to block free cysteines. Proteins were precipitated with cold acetone, washed twice, resuspended in HENS buffer (1% SDS), and incubated with 0.4 mM biotin-HPDP and 5 mM sodium ascorbate at room temperature for 1 h in the dark. Biotinylated proteins were purified using streptavidin-agarose beads, separated by SDS-PAGE, and detected by immunoblotting.

#### SPR analysis

SPR analysis was performed using the Biacore 8K system (Cytiva) to assess the binding affinity of β-cat-Oxazole to β-catenin. CM5 sensor chips were activated with N-hydroxysulfosuccinimide and 1-(3-dimethylaminopropyl)-3-ethylcarbodiimide methiodide (EDC), followed by immobilization of purified β-catenin (50 μg/mL in sodium acetate, pH 5.0). The surface was blocked with ethanolamine to prevent nonspecific binding. Gradient concentrations of β-cat-Oxazole were injected over the immobilized protein at a flow rate of 30 μL/min in a running buffer containing 0.05% Tween 20 in PBS. SPR data were collected and analyzed using Biacore Insight evaluation software (Cytiva, Marlborough, MA, USA).

#### Integrated transcriptomic analysis

RNA-seq data from intestinal epithelium-specific *Zdhhc5*-knockout mice, *CTNNB1*-knockout HCT116 cells, and *ZDHHC5*-knockdown DLD1 cells were analyzed separately. Differentially expressed genes (DEGs) were identified with a threshold of |log2 fold change (FC)| > 1 and *p* < 0.05. The lists of significantly downregulated DEGs from each model were then intersected with a published ferroptosis-related gene set^37^ to identify common candidate targets.

#### ScRNA-seq and spatial transcriptome analysis

The GSE132465 CRC scRNA-Seq dataset and associated cell metadata were retrieved from a previous study.^54^ The CRC spatial transcriptome dataset was obtained from another study.^38^ ScRNA-Seq and spatial transcriptome data processing and visualization were conducted following methodologies outlined in our previous publication.^55^

#### Atomic modeling analysis

(i) 3D protein structure prediction: The complex structures of ZDHHC5 and β-catenin were predicted using the AlphaFold3 multimer program.^56^ The conformation with the highest confidence score was selected for subsequent molecular dynamics simulation and analysis.
(ii) Molecular dynamics simulations: The GROMACS package (version 2023.03; https://www.sciencedirect.com/science/article/pii/S2352711015000059) was utilized to perform conventional molecular dynamics simulations, aiming to investigate conformational changes in the ZDHHC5-β-catenin complex. The Amber14sb force field^57^ was employed for protein parameterization, and the TIP3P water model (https://www.scopus.com/pages/publications/0004016501) was used for solvent representation. The ZDHHC5-β-catenin complex was solvated in an octahedral water box, and the system charge was neutralized by adding 0.150 M NaCl. Initially, energy minimization was performed using the steepest descent algorithm for 50,000 steps to relieve steric clashes. Subsequently, position restraints were applied to heavy atoms during 50,000-step NVT (constant number of particles, volume, and temperature) and NPT (constant number of particles, pressure, and temperature) equilibration phases. The system temperature was maintained at 300 K, and the pressure was kept at 1 bar. Following equilibration, an unrestrained 100-ns production simulation was conducted, with trajectory coordinates and energy parameters saved every 20 ps. Interaction patterns and dynamic trajectories were visualized and analyzed using ChimeraX.^58^
(iii) Free energy calculations and residue decomposition: The MM-GBSA method^59^ is widely used for estimating binding free energies in drug discovery research. In this study, MM-GBSA calculations were performed using gmx_MMPBSA,^60^ a GROMACS-compatible tool for MM-PBSA/MM-GBSA analyses. To elucidate the molecular basis of ZDHHC5-β-catenin interactions, gmx_MMPBSA was further employed to decompose the binding free energy into individual residue contributions.

High-throughput virtual screening for ZDHHC5/β-catenin inhibitors using dynamic complex structures and cascade docking.

(i) Protein structure preparation and preprocessing: Initial protein structures were preprocessed using the Protein Preparation Wizard (Schrödinger). This procedure included the assignment of bond orders, addition of missing hydrogen atoms, and optimization of hydrogen-bonding networks with PROPKArot physiological pH 7.0. Potential binding sites at the interface between ZDHHC5 and β-catenin were predicted using the SiteMap module. Subsequently, docking grids were generated with the Receptor Grid Generation tool in Glide. A cubic bounding box of 20 × 20 × 20 Å, centered on the identified binding site, was defined to serve as the precise spatial framework for all subsequent molecular docking calculations.
(ii) Small molecule database preparation: The screening database was obtained from ChemDiv (https://www.chemdiv.com/) and processed using Schrödinger’s LigPrep module. Preprocessing steps included desalting, addition of hydrogen atoms, generation of possible ionization states at a target pH of 7.0 ± 2.0 using Epik, enumeration of tautomers and stereoisomers, and energy minimization employing the OPLS4 force field.
(iii) Virtual screening: An initial ADMET (Absorption, Distribution, Metabolism, Excretion, and Toxicity) filter was applied to the database molecules using Schrödinger’s QuickProp module, retaining only compounds that satisfied Lipinski’s Rule of Five and contained no reactive functional groups. High-throughput virtual screening (HTVS) was then performed using the Glide retain, with GlideScore (GScore) serving as the primary scoring function. The top 10% of compounds ranked by HTVS score were retained for subsequent Standard Precision (SP) docking. Following this, the top 10%com compounds from the SP docking stage, which employed the more rigorous Glide SP scoring function, were subjected to Extra Precision (XP) docking. The XP docking stage applied the Glide XP scoring function, which imposes stricter penalties for desolvation and steric clashes. The top 10% of compounds from XP docking were further analyzed using the Prime MM-GBSA module and the VSGB 2.0 solvation model to calculate binding free energies. Based on the MM-GBSA scores, the top 30 compoundscompo prioritized for procurement and subsequent experimental validation.
(iiii) Molecular dynamics simulation analysis: To refine the binding pose of the lead compound, β-cat-Oxazole, in complex with β-catenin, molecular dynamics simulations were performed using Desmond. The OPLS4olecul field was used for the protein and ligand, with water molecules modeled explicitly using the TIP3P model. The protein-ligand complex was solvated in a cubic water box, maintaining a minimum distance of 10 Å from the protein surface to the box edge. The system was neutralized with 0.150 M sodium and chloride ions. Energy minimization was conducted for 50,000 steps using the steepest descent algorithm. This was followed by a 50,000-step equilibration phase under NVT (constant number of particles, volume, and temperature) and NPT (constant number of particles, pressure, and temperature) ensembles, with harmonic restraints applied to protein heavy atoms. System temperature and pressure were maintained at 300 K and 1 bar using the Nosé-Hoover chain thermostat and the Martyna-Tobias-Klein barostat, respectively. Finally, a 100 ns production simulation was performed without restraints, with trajectory frames saved every 10 ps for analysis.

### Quantification and statistical analysis

Data analysis was performed using GraphPad Prism 9 and R software. Survival analysis was conducted using Kaplan–Meier curves and the log rank test. Pearson’s correlation coefficient was used to assess the relationship between variables. Statistical significance was set at *p* < 0.05 and determined by the two-tailed Student’s *t* test. Data are presented as mean ± standard deviation (SD).

## Supplemental information

Document S1. Figures S1–S14

Table S1. Clinical characteristics of CRC patients for β-catenin palmitoylation analysis

Table S2. Clinical information of CRC patients in TMA

Table S3. Clinical characteristics of CRC patients for SLC7A11 expression analysis

Table S4. List of primer and shRNA sequences used in this study

Document S2. Article plus supplemental information

## References

[1] Ruiz de Galarreta M., Bresnahan E., Molina-Sánchez P., Lindblad K.E., Maier B., Sia D., Puigvehi M., Miguela V., Casanova-Acebes M., Dhainaut M. (2019). β-Catenin Activation Promotes Immune Escape and Resistance to Anti-PD-1 Therapy in Hepatocellular Carcinoma. Cancer Discov..

[2] Muzny D.M., Bainbridge M.N., Chang K., Dinh H.H., Drummond J.A., Fowler G., Kovar C.L., Lewis L.R., Morgan M.B., Newsham I.F. (2012). Comprehensive molecular characterization of human colon and rectal cancer. Nature.

[3] Yuan S., Tao F., Zhang X., Zhang Y., Sun X., Wu D. (2020). Role of Wnt/β-Catenin Signaling in the Chemoresistance Modulation of Colorectal Cancer. Biomed Res. Int..

[4] Wang Y., Zheng L., Shang W., Yang Z., Li T., Liu F., Shao W., Lv L., Chai L., Qu L. (2022). Wnt/beta-catenin signaling confers ferroptosis resistance by targeting GPX4 in gastric cancer. Cell Death Differ..

[5] Chocarro-Calvo A., García-Martínez J.M., Ardila-González S., De la Vieja A., García-Jiménez C. (2013). Glucose-Induced β-Catenin Acetylation Enhances Wnt Signaling in Cancer. Mol. Cell.

[6] Wang B., Wang T., Zhu H., Yan R., Li X., Zhang C., Tao W., Ke X., Hao P., Qu Y. (2022). Neddylation is essential for β-catenin degradation in Wnt signaling pathway. Cell Rep..

[7] Guo Y., Tian J., Guo Y., Wang C., Chen C., Cai S., Yu W., Sun B., Yan J., Li Z. (2023). Oncogenic KRAS effector USP13 promotes metastasis in non-small cell lung cancer through deubiquitinating b-catenin. Cell Rep..

[8] Miao Z., Zhao X., Liu X. (2023). Hypoxia induced β-catenin lactylation promotes the cell proliferation and stemness of colorectal cancer through the wnt signaling pathway. Exp. Cell Res..

[9] Liu J., Ren G., Li K., Liu Z., Wang Y., Chen T., Mu W., Yang X., Li X., Shi A. (2022). The Smad4-MYO18A-PP1A complex regulates β-catenin phosphorylation and pemigatinib resistance by inhibiting PAK1 in cholangiocarcinoma. Cell Death Differ..

[10] Zhang Q., Yang X., Wu J., Ye S., Gong J., Cheng W.M., Luo Z., Yu J., Liu Y., Zeng W. (2023). Reprogramming of palmitic acid induced by dephosphorylation of ACOX1 promotes β-catenin palmitoylation to drive colorectal cancer progression. Cell Discov..

[11] Fang C., Zhang X., Zhang L., Gao X., Yang P., Lu H. (2016). Identification of Palmitoylated Transitional Endoplasmic Reticulum ATPase by Proteomic Technique and Pan Antipalmitoylation Antibody. J. Proteome Res..

[12] Yang W., Di Vizio D., Kirchner M., Steen H., Freeman M.R. (2010). Proteome scale characterization of human S-acylated proteins in lipid raft-enriched and non-raft membranes. Mol. Cell. Proteomics.

[13] Zhang Y., Chidiac R., Delisle C., Gratton J.P. (2017). Endothelial NO Synthase-Dependent S-Nitrosylation of β-Catenin Prevents Its Association with TCF4 and Inhibits Proliferation of Endothelial Cells Stimulated by Wnt3a. Mol. Cell Biol..

[14] Gu M., Jiang H., Tan M., Yu L., Xu N., Li Y., Wu H., Hou Q., Dai C. (2023). Palmitoyltransferase DHHC9 and acyl protein thioesterase APT1 modulate renal fibrosis through regulating β-catenin palmitoylation. Nat. Commun..

[15] Chong X., Zhu L., Yu D., Chen S., Wang G., Yu Q., Ma X., Xu J., Chen H., An H. (2023). ZDHHC9 promotes colon tumor growth by inhibiting effector T cells. Oncol. Lett..

[16] Li W., Liu J., Yu T., Lu F., Miao Q., Meng X., Xiao W., Yang H., Zhang X. (2024). ZDHHC9-mediated Bip/GRP78 S-palmitoylation inhibits unfolded protein response and promotes bladder cancer progression. Cancer Lett..

[17] Li Z., Jiang D., Liu F., Li Y. (2023). Involvement of ZDHHC9 in lung adenocarcinoma: regulation of PD-L1 stability via palmitoylation. In Vitro Cell Dev-An.

[18] Lin Z., Huang K., Guo H., Jia M., Sun Q., Chen X., Wu J., Yao Q., Zhang P., Vakal S. (2023). Targeting ZDHHC9 potentiates anti-programmed death-ligand 1 immunotherapy of pancreatic cancer by modifying the tumor microenvironment. Biomed. Pharmacother..

[19] Chen L., Xing X., Zhu Y., Chen Y., Pei H., Song Q., Li J., Zhang P. (2024). Palmitoylation alters LDHA activity and pancreatic cancer response to chemotherapy. Cancer Lett..

[20] Liu Q., Li W., Xie M., Yang M., Xu M., Yang L., Sun M., Peng Y., Gao L. (2025). ZDHHC9 as a Potential Biomarker for Prognostic Evaluation and Diagnostic Identification in BRCA, CESC, HNSC, and KIRP Tumors. Eur. J. Cancer Care.

[21] Apetoh L., Ghiringhelli F., Tesniere A., Obeid M., Ortiz C., Criollo A., Mignot G., Maiuri M.C., Ullrich E., Saulnier P. (2007). Toll-like receptor 4-dependent contribution of the immune system to anticancer chemotherapy and radiotherapy. Nat. Med..

[22] Cen B., Wei J., Wang D., Xiong Y., Shay J.W., DuBois R.N. (2021). Mutant APC promotes tumor immune evasion via PD-L1 in colorectal cancer. Oncogene.

[23] Ko P.J., Woodrow C., Dubreuil M.M., Martin B.R., Skouta R., Bassik M.C., Dixon S.J. (2019). A ZDHHC5-GOLGA7 Protein Acyltransferase Complex Promotes Nonapoptotic Cell Death. Cell Chem. Biol..

[24] Stix R., Song J., Banerjee A., Faraldo-Gómez J.D. (2020). DHHC20 Palmitoyl-Transferase Reshapes the Membrane to Foster Catalysis. Biophys. J..

[25] Lee C.J., Stix R., Rana M.S., Shikwana F., Murphy R.E., Ghirlando R., Faraldo-Gómez J.D., Banerjee A. (2022). Bivalent recognition of fatty acyl-CoA by a human integral membrane palmitoyltransferase. Proc. Natl. Acad. Sci. USA.

[26] Mesquita F.S., Abrami L., Linder M.E., Bamji S.X., Dickinson B.C., van der Goot F.G. (2024). Mechanisms and functions of protein S-acylation. Nat. Rev. Mol. Cell Bio..

[27] Kahlson M.A., Ritho J., Gomes J.V., Wang H., Butterwick J.A., Dixon S.J. (2025). Functional dissection of the zDHHC palmitoyltransferase 5-golgin A7 palmitoylation complex. J. Biol. Chem..

[28] Jung Y.S., Wang W., Jun S., Zhang J., Srivastava M., Kim M.J., Lien E.M., Shang J., Chen J., McCrea P.D. (2018). Deregulation of CRAD-controlled cytoskeleton initiates mucinous colorectal cancer via β-catenin. Nat. Cell Biol..

[29] Liu E., Zhou Q., Xie A.J., Li X., Li M., Ye J., Li S., Ke D., Wang Q., Xu Z.P. (2020). Tau acetylates and stabilizes β-catenin thereby promoting cell survival. EMBO Rep..

[30] Zhang T., Liu S., Yang P., Han C., Wang J., Liu J., Han Y., Yu Y., Cao X. (2009). Fibronectin maintains survival of mouse natural killer (NK) cells via CD11b/Src/β-catenin pathway. Blood.

[31] Zhang Q., Cui K., Kong Y., Yu J., Luo Z., Yang X., Gong L., Xie Y., Lin J., Liu C. (2025). Targeting both the enzymatic and non-enzymatic functions of DHODH as a therapeutic vulnerability in c-Myc-driven cancer. Cell Rep..

[32] Ilangovan R., Marshall W.L., Hua Y., Zhou J. (2003). Inhibition of apoptosis by Z-VAD-fmk in SMN-depleted S2 cells. J. Biol. Chem..

[33] Cao L., Mu W. (2021). Necrostatin-1 and necroptosis inhibition: Pathophysiology and therapeutic implications. Pharmacol. Res..

[34] Shi Y., Tao M., Ma X., Hu Y., Huang G., Qiu A., Zhuang S., Liu N. (2020). Delayed treatment with an autophagy inhibitor 3-MA alleviates the progression of hyperuricemic nephropathy. Cell Death Dis..

[35] Zhao Y., Li M., Yao X., Fei Y., Lin Z., Li Z., Cai K., Zhao Y., Luo Z. (2020). HCAR1/MCT1 Regulates Tumor Ferroptosis through the Lactate-Mediated AMPK-SCD1 Activity and Its Therapeutic Implications. Cell Rep..

[36] Li J., Liu J., Zhou Z., Wu R., Chen X., Yu C., Stockwell B., Kroemer G., Kang R., Tang D. (2023). Tumor-specific GPX4 degradation enhances ferroptosis-initiated antitumor immune response in mouse models of pancreatic cancer. Sci. Transl. Med..

[37] Zhou N., Yuan X., Du Q., Zhang Z., Shi X., Bao J., Ning Y., Peng L. (2023). FerrDb V2: update of the manually curated database of ferroptosis regulators and ferroptosis-disease associations. Nucleic Acids Res..

[38] Wu Y., Yang S., Ma J., Chen Z., Song G., Rao D., Cheng Y., Huang S., Liu Y., Jiang S. (2022). Spatiotemporal Immune Landscape of Colorectal Cancer Liver Metastasis at Single-Cell Level. Cancer Discov..

[39] Du L., Lee J.H., Jiang H., Wang C., Wang S., Zheng Z., Shao F., Xu D., Xia Y., Li J. (2020). β-Catenin induces transcriptional expression of PD-L1 to promote glioblastoma immune evasion. J. Exp. Med..

[40] Zhang Z., Zhou H., Gu W., Wei Y., Mou S., Wang Y., Zhang J., Zhong Q. (2024). CGI1746 targets σ(1)R to modulate ferroptosis through mitochondria-associated membranes. Nat. Chem. Biol..

[41] Liu H., Wen S., Xu C., Kang X., Kong E. (2025). Mechanisms and functional implications of ZDHHC5 in cellular physiology and disease. J. Lipid Res..

[42] Shimell J.J., Globa A., Sepers M.D., Wild A.R., Matin N., Raymond L.A., Bamji S.X. (2021). Regulation of hippocampal excitatory synapses by the Zdhhc5 palmitoyl acyltransferase. J. Cell Sci..

[43] Woodley K.T., Collins M.O. (2021). Regulation and function of the palmitoyl-acyltransferase ZDHHC5. FEBS J..

[44] Xiu C., Ji K., Zhao G., Chen J., Yang Y. (2025). The emerging role of palmitoyl acyltransferase zDHHC5 in health and disease: A review. Int. J. Biol. Macromol..

[45] O'Leary B., Finn R.S., Turner N.C. (2016). Treating cancer with selective CDK4/6 inhibitors. Nat. Rev. Clin. Oncol..

[46] Haupenthal J., Bihrer V., Korkusuz H., Kollmar O., Schmithals C., Kriener S., Engels K., Pleli T., Benz A., Canamero M. (2012). Reduced Efficacy of the Plk1 Inhibitor BI 2536 on the Progression of Hepatocellular Carcinoma due to Low Intratumoral Drug Levels. Neoplasia.

[47] Wang Z., Li Y., Sun S., Chen J., Zheng F., Hu Z., Zhang J., Liu B., Liu M. (2025). ZDHHC5 deficiency impairs spermatogenesis and causes male infertility in mice. Reproduction.

[48] Jung Y.S., Jun S., Kim M.J., Lee S.H., Suh H.N., Lien E.M., Jung H.Y., Lee S., Zhang J., Yang J.I. (2018). TMEM9 promotes intestinal tumorigenesis through vacuolar-ATPase-activated Wnt/β-catenin signalling. Nat. Cell Biol..

[49] Domon B., Aebersold R. (2006). Review - Mass spectrometry and protein analysis. Science.

[50] Zhang Q., Li X., Cui K., Liu C., Wu M., Prochownik E.V., Li Y. (2020). The MAP3K13-TRIM25-FBXW7α axis affects c-Myc protein stability and tumor development. Cell Death Differ..

[51] Cui K., Wang K., Huang Z. (2024). Ferroptosis and the tumor microenvironment. J. Exp. Clin. Cancer Res..

[52] Drisdel R.C., Alexander J.K., Sayeed A., Green W.N. (2006). Assays of protein palmitoylation. Methods.

[53] Jaffrey S.R., Snyder S.H. (2001). The biotin switch method for the detection of S-nitrosylated proteins. Sci. STKE.

[54] Lee H.O., Hong Y., Etlioglu H.E., Cho Y.B., Pomella V., Van den Bosch B., Vanhecke J., Verbandt S., Hong H., Min J.W. (2020). Lineage-dependent gene expression programs influence the immune landscape of colorectal cancer. Nat. Genet..

[55] Cui K., Liu B., Gong L., Wan Q., Tang H., Gong Z., Shen R., Wang C., Zhang Q., Li Q. (2025). Targeting ribosomes reprograms the tumour microenvironment and augments cancer immunotherapy. Br. J. Cancer.

[56] Abramson J., Adler J., Dunger J., Evans R., Green T., Pritzel A., Ronneberger O., Willmore L., Ballard A.J., Bambrick J. (2024). Accurate structure prediction of biomolecular interactions with AlphaFold 3. Nature.

[57] Nguyen H., Maier J., Huang H., Perrone V., Simmerling C. (2014). Folding Simulations for Proteins with Diverse Topologies Are Accessible in Days with a Physics-Based Force Field and Implicit Solvent. J. Am. Chem. Soc..

[58] Goddard T.D., Huang C.C., Meng E.C., Pettersen E.F., Couch G.S., Morris J.H., Ferrin T.E. (2018). UCSF ChimeraX: Meeting modern challenges in visualization and analysis. Protein Sci..

[59] Genheden S., Ryde U. (2015). The MM/PBSA and MM/GBSA methods to estimate ligand-binding affinities. Expert Opin. Drug Dis..

[60] Valdés-Tresanco M.S., Valdés-Tresanco M.E., Valiente P.A., Moreno E. (2021). gmx_MMPBSA: A New Tool to Perform End-State Free Energy Calculations with GROMACS. J. Chem. Theory Comput..

