# Supplementary material for: Druggable β-catenin palmitoyl-switch coordinates immune evasion via immunogenic ferroptosis resistance and PD-L1-mediated immunosuppression
Source: Cell Rep Med. 2026 May 28;7(6):102837. doi: 10.1016/j.xcrm.2026.102837 (PMC13294000; doi:10.1016/j.xcrm.2026.102837)
Supplement: Document S1. Figures S1–S14 [file mmc1.pdf]

Cell Reports Medicine, Volume 7

Supplemental information

Druggable β-catenin palmitoyl-switch coordinates immune evasion via immunogenic ferroptosis resistance and PD-L1-mediated immunosuppression

Qiang Zhang, Yue Kong, Yinglin Long, Xue Li, Liang Wang, Zhanhao Luo, Xiaoya Yang, Yanchun Xie, Quanwei Yu, Jing Yu, Dayi Liang, Wenhao Yuan, Xiaomin Cheng, Yaqi Su, Kaisa Cui, Guobao Tian, Zhen He, and Ping Lan

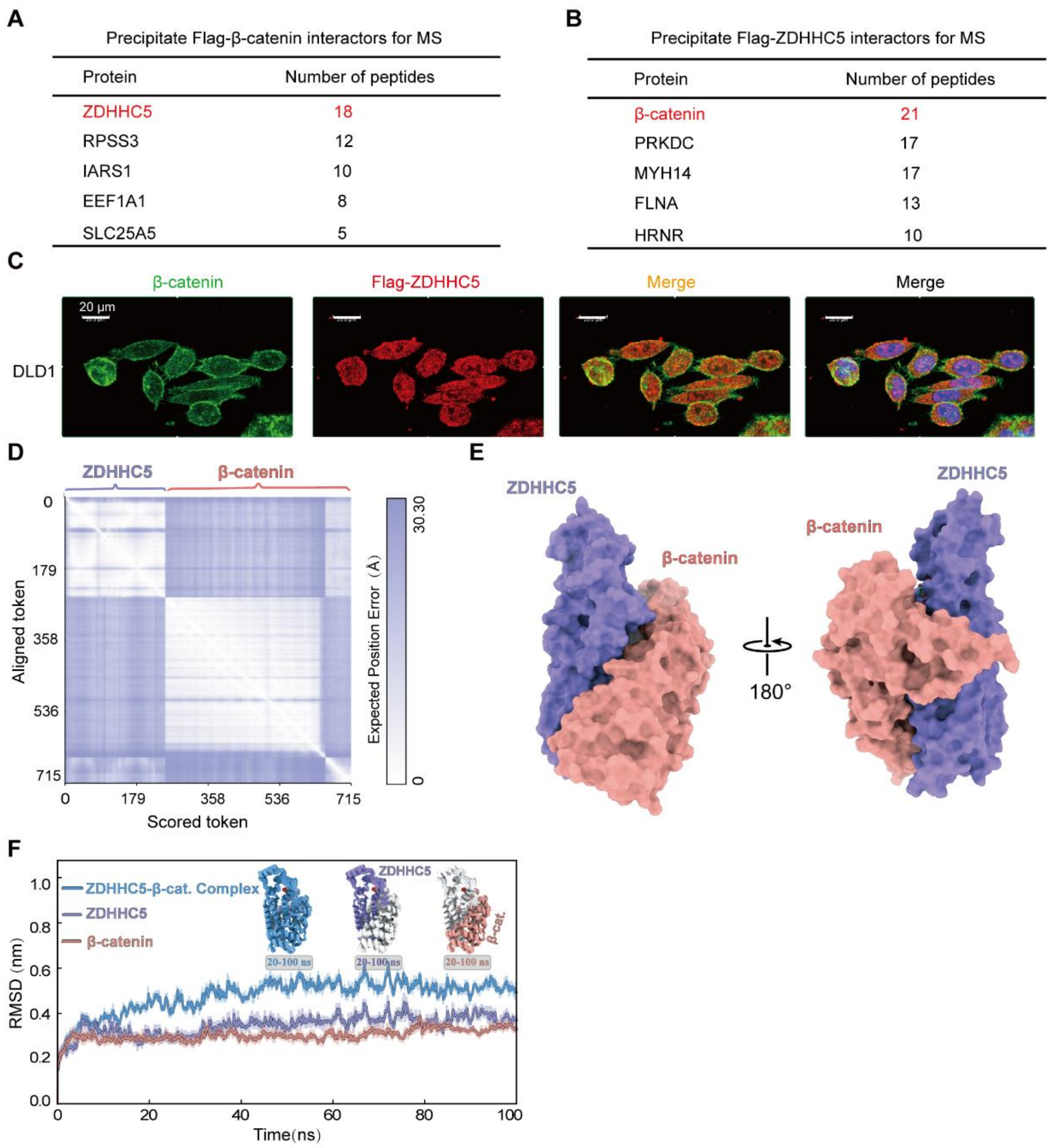

Figure S1 Identification and initial characterization of the interaction between ZDHHC5 and β-catenin. Related to Figure 1

(A and B) MS analysis identifies ZDHHC5 and β-catenin as mutual interactors. List of top proteins co-immunoprecipitated with Flag-β-catenin in HEK293T cells (A). Reciprocal MS analysis of proteins co-immunoprecipitated with Flag-ZDHHC5 (B).

(C) Subcellular co-localization of ZDHHC5 and β-catenin. Immunofluorescence staining of DLD1 CRC cells showing the localization of β-catenin (green) and Flag-ZDHHC5 (red). Nuclei are counterstained with DAPI (blue).

(D) Distance matrix between the amino acids of ZDHHC5 and β-catenin.

(E) AlphaFold3 modeled structure of ZDHHC5 and β-catenin.

(F) Root mean square deviation (RMSD) of the ZDHHC5-β-catenin complex during 100 ns molecular dynamics simulation.

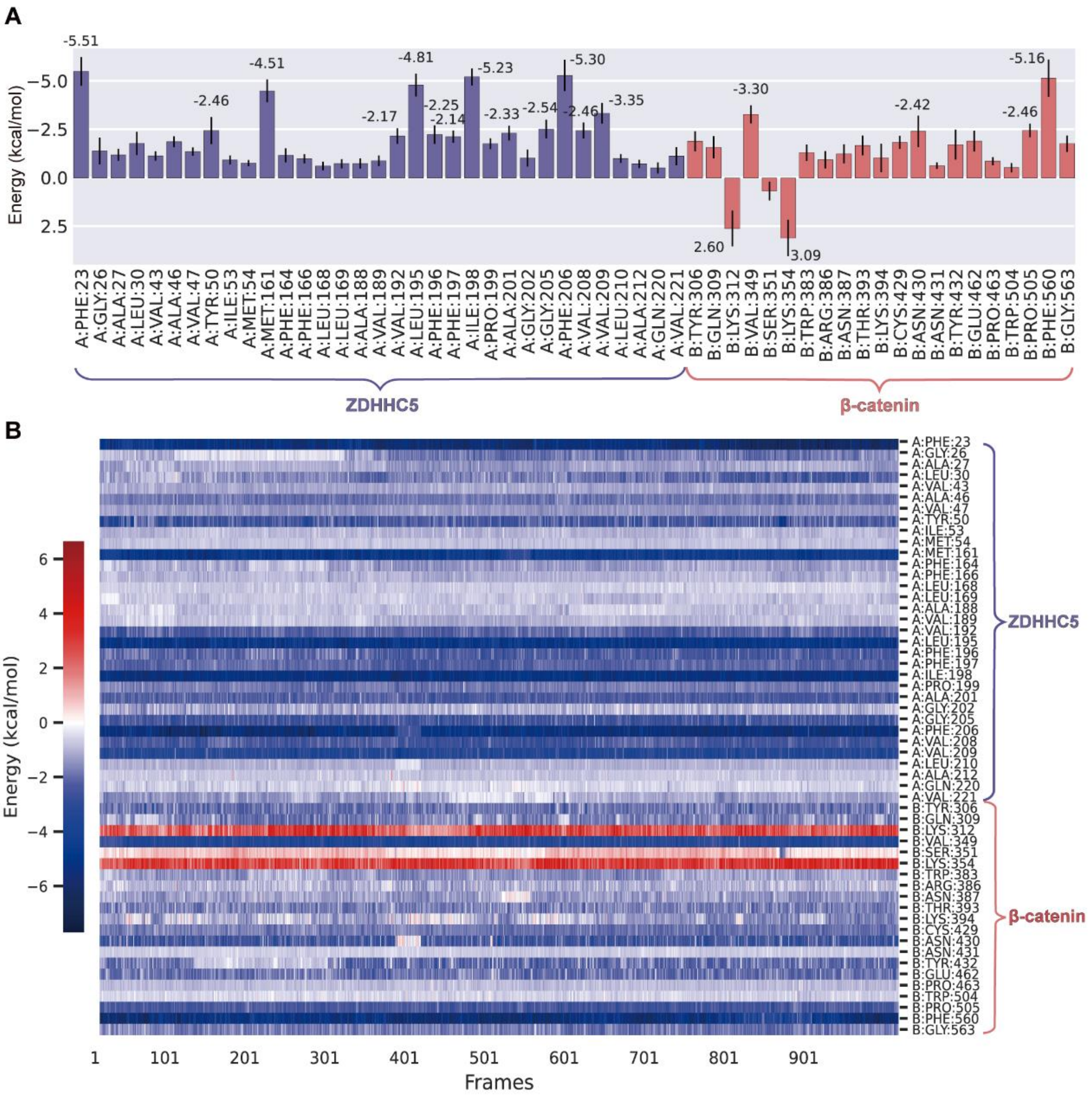

Figure S2 Computational analysis of residue contributions to the ZDHHC5-β-catenin interface. Related to Figure 1

(A) Decomposition of the binding free energy between ZDHHC5 and β-catenin into contributions from interacting surface amino acids.

(B) Decomposition of the binding free energy between ZDHHC5 and β-catenin into contributions from interacting surface amino acids at each frame.

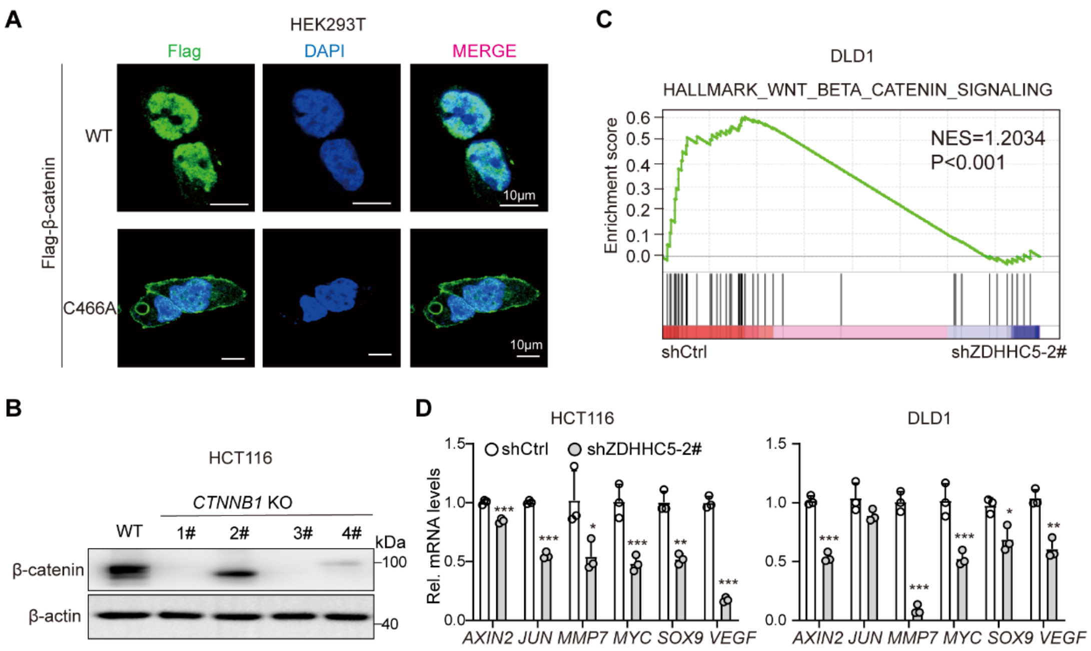

Figure S3 ZDHHC5 activates β-catenin transcriptional activity. Related to Figure 3

(A) Subcellular localization of Flag-tagged β-catenin variants in HEK293T cells. WT Flag-β-catenin or its C466A mutant was transfected into HEK293T cells, followed by immunofluorescence analysis.

(B) Immunoblot validation of *CTNNB1* knockout in HCT116 cells.

(C) Gene set enrichment analysis (GSEA) of the HALLMARK_WNT_BETA_CATENIN_SIGNALING pathway in DLD1 cells upon *ZDHHC5* knockdown.

(D) qRT-PCR analysis of canonical Wnt/β-catenin target genes in HCT116 and DLD1 cells upon *ZDHHC5* knockdown .

Data are presented as mean ± SD; Statistical significance was determined by Student's t test; * $P < 0.05$, ** $P < 0.01$, *** $P < 0.001$.

A

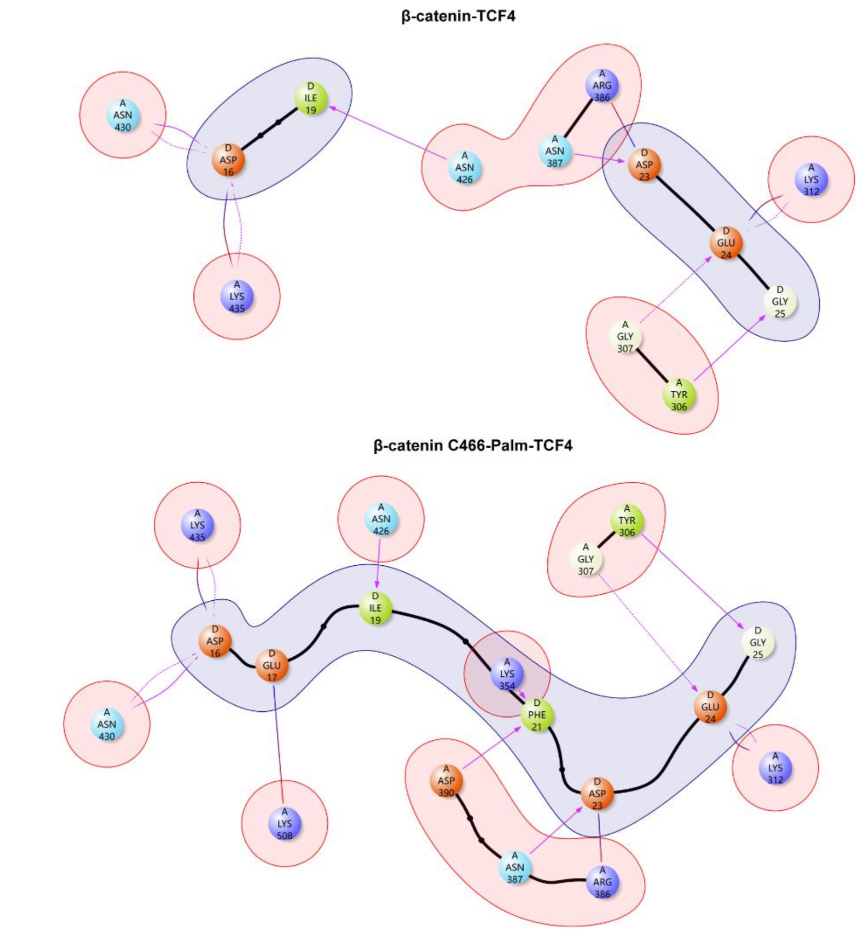

B

β-catenin-TCF4 residue interaction

β-catenin	TCF4	Distance (Å)	Specific Interactions
A: LYS 435	D:ASP 16	2.4	1xhb, 1xsalt bridge to D:ASP 16
A: ASN 430	D:ASP 16	2.0	2xhb to D:ASP 16
A: ASN 426	D:ILE 19	1.9	2xhb to D:ILE 19
A: ASN 387	D:ASP 23	1.9	2xhb to D:ASP 23
A: ARG 386	D:ASP 23	2.6	1xsalt bridge to D:ASP 23
A: LYS 312	D:GLU 24	2.4	1xhb, 1xsalt bridge to D:GLU 24
A: LYS 307	D:GLU 24	2.1	1xhb to D:GLU 24
A: TYR 306	D:GLY 25	2.1	1xhb to D:GLY 25

β-catenin C466-Palm-TCF4 residue interaction

β-catenin	TCF4	Distance (Å)	Specific Interactions
A: **LYS 508**	D:**GLU 17**	2.7	**1xsalt bridge to D:GLU 17**
A: LYS 435	D:ASP 16	2.4	2xhb, 1xsalt bridge to D:ASP 16
A: ASN 430	D:ASP 16	2.1	2xhb to D:ASP 16
A: ASN 426	D:ILE 19	1.9	2xhb to D:ILE 19
A: **ASP 390**	D:**PHE 21**	2.5	**1xhb to D:PHE 21**
A: ASN 387	D:ASP 23	1.9	2xhb to D:ASP 23
A: ARG 386	D:ASP 23	2.6	1xsalt bridge to D:ASP 23
A: **LYS 354**	D:**PHE 21**	2.5	**1xhb to D:PHE 21**
A: LYS 312	D:GLU 24	2.4	1xhb,1xsalt bridge to D:GLU 24
A: GLY 307	D:GLU 24	2.1	1xhb to D:GLU 24
A: TYR 306	D:GLY 25	2.1	1xhb to D:GLY 25

Figure S4 Computational modeling of the β-catenin/TCF4 complex in the C466 non-palmitoylated and palmitoylated states. Related to Figure 3

(A) Predicted interaction network in the canonical, non-palmitoylated β-catenin (**upper**) and β-catenin C466 palmitoylation (**lower**) state. Key interactions are shown,

including salt bridges and hydrogen bonds.

(B) The tables detail the predicted intermolecular interactions between β-catenin and TCF4 residues in the non-palmitoylated (**panel A upper**) and (**panel A lower**) C466-palmitoylated states, derived from structural modeling. Notably, a salt bridge forms between β-catenin Lys508 and TCF4 Glu17 (2.7Å). Additionally, new hydrogen bonds are established between β-catenin Asp390/Lys354 and TCF4 Phe21 (both 2.5 Å). hb: hydrogen bond; Distance values are in Ångströms.

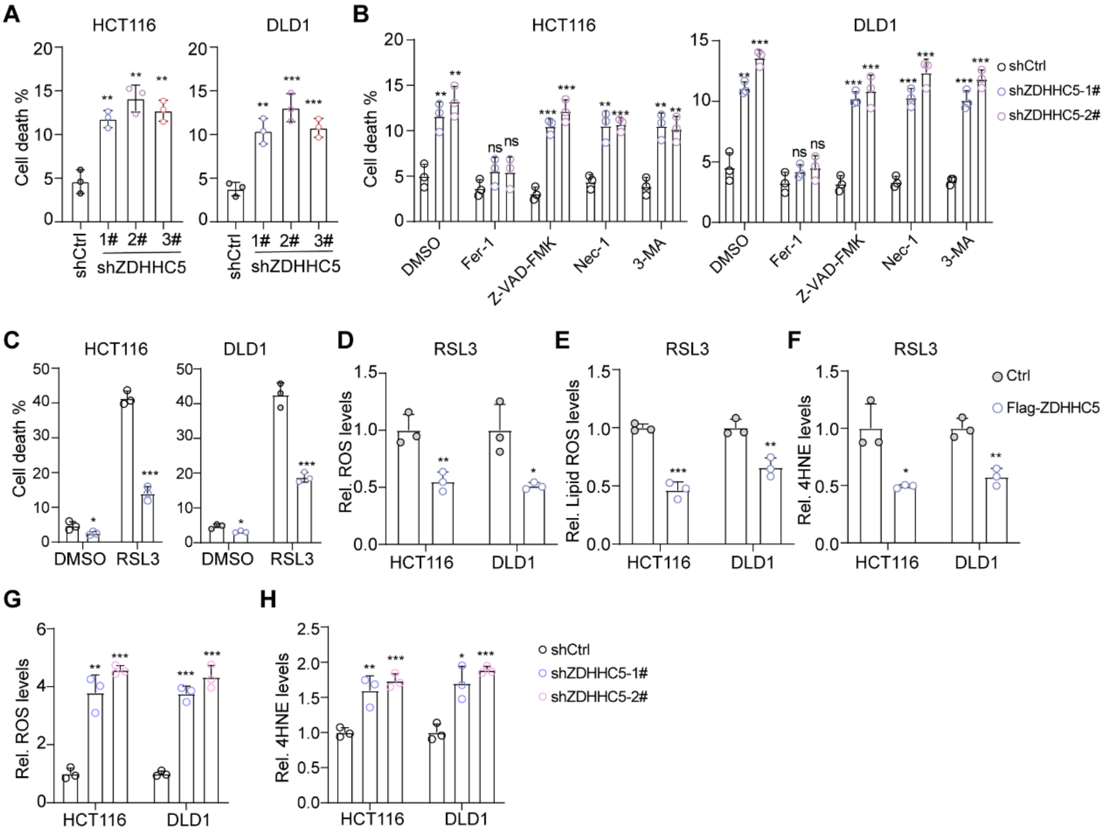

Figure S5 ZDHHC5 promotes resistance to ferroptosis. Related to Figure 4.

(A) Cell death of CRC cell upon *ZDHHC5* knockdown. Stable *ZDHHC5* knockdown HCT116 and DLD1 cells were subjected to trypan blue staining assays.

(B) Cell death induced by *ZDHHC5* knockdown is rescued by ferroptosis inhibitor.

ZDHHC5-knockdown HCT116 and DLD1 cells were treated with ferroptosis inhibitor (10 μM Ferrostatin-1), apoptosis inhibitor (10 μM Z-VAD-FMK), necrosis inhibitor (10 μM Nec-1), or autophagy inhibitor (1 mM 3-MA), followed by trypan blue staining assays.

(C) ZDHHC5 inhibits RSL3-induced ferroptosis. HCT116 and DLD1 cells overexpressing ZDHHC5 were treated with RSL3 (3 μM) for 48 hours, followed by trypan blue staining assays.

(D-F) Overexpression of ZDHHC5 reduces levels of ROS (**D**), Lipid ROS (**E**), and 4-HNE (**F**) in HCT116 and DLD1 cells.

(G and H) Knockdown of *ZDHHC5* increases ROS (**G**) and 4-HNE (**H**) levels in HCT116 and DLD1 cells.

Data are presented as mean ± SD; Statistical significance was determined by Student's t test; * $P < 0.05$, ** $P < 0.01$, *** $P < 0.001$.

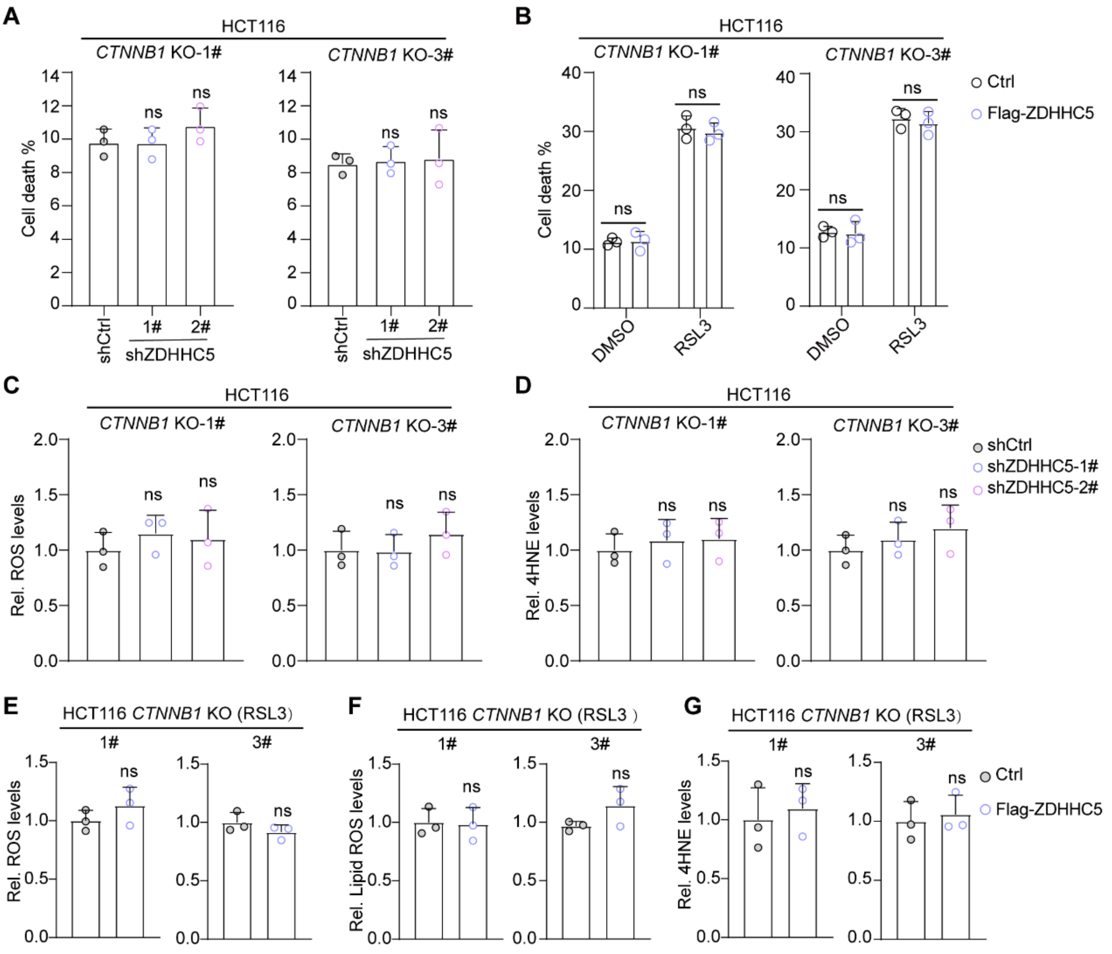

Figure S6 ZDHHC5 promotes ferroptosis resistance dependent on β-catenin. Related to Figure 4.

(A) Knockdown of *ZDHHC5* in *CTNNB1* knockout HCT116 cells, followed by trypan blue staining assays.

(B) Overexpression of ZDHHC5 in *CTNNB1* knockout HCT116 cells treated with RSL3 (3 μM), followed by trypan blue staining assays.

(C and D) ROS (**C**) and 4-HNE (**D**) levels were measured in *CTNNB1* knockout HCT116 cells after *ZDHHC5* knockdown.

(E-G) ROS (**E**), Lipid ROS (**F**), and 4-HNE (**G**) levels were measured in *CTNNB1* knockout HCT116 cells after overexpression of ZDHHC5 followed by RSL3

treatment (3 μM).

Data are presented as mean ± SD; Statistical significance was determined by Student's t test; "ns" stands for "not significant".

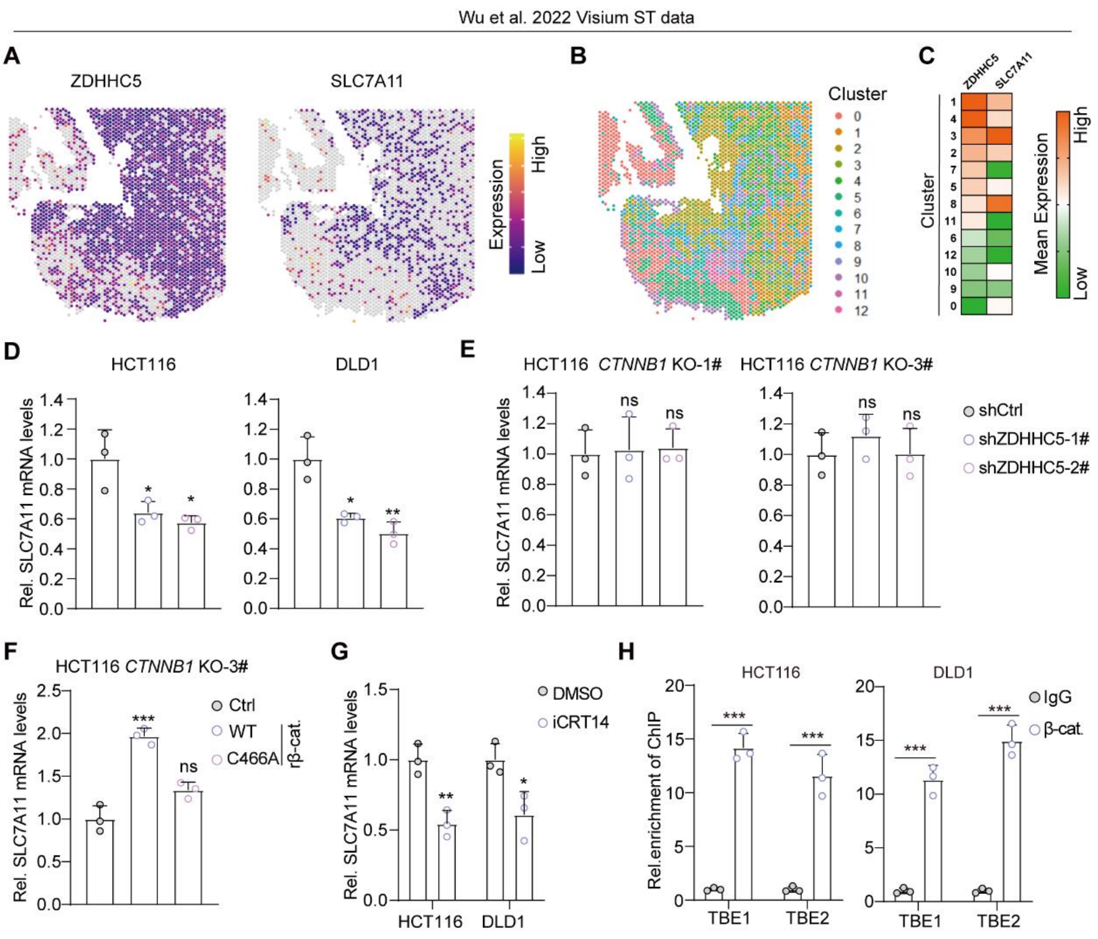

Figure S7 ZDHHC5 regulates SLC7A11 expression through β-catenin C466 palmitoylation. Related to Figure 4.

(A) Spatial expression patterns of *ZDHHC5* and *SLC7A11* genes in CRC tissue sections, analyzed via Visium spatial transcriptomics (data from Wu et al., 2022).

(B) Spatial clustering of tissue sections based on transcriptomic profiles, analyzed using Visium spatial transcriptomics (data from Wu et al., 2022).

(C) Heatmap showing mean expression levels of *ZDHHC5* and *SLC7A11* across

spatial clusters defined in panel **B**.

(D) Knockdown of *ZDHHC5* decreases *SLC7A11* expression. Stable *ZDHHC5* knockdown HCT116 and DLD1 cells were analyzed by qPCR.

(E) ZDHHC5 does not affect *SLC7A11* expression in HCT116 *CTNNB1* knockout cells.

(F) β-catenin C466 palmitoylation is required for SLC7A11 expression. *CTNNB1* knockout HCT116 cells were re-expressed with either WT β-catenin or C466A mutant, followed by qPCR analysis.

(G) Inhibition of β-catenin/TCF4 interaction with iCRT14 decreases *SLC7A11* expression. HCT116 and DLD1 cells were treated with iCRT14(100μM) for 24h, followed by qPCR analysis.

(H) β-catenin binds to the TBE region of the SLC7A11 promoter. Chromatin immunoprecipitation assays were performed on HCT116 and DLD1 cells using an anti-β-catenin antibody.

Data are presented as mean ± SD; Statistical significance was determined by Student's t test; * P < 0.05, ** P < 0.01, *** P < 0.001; "ns" stands for "not significant".

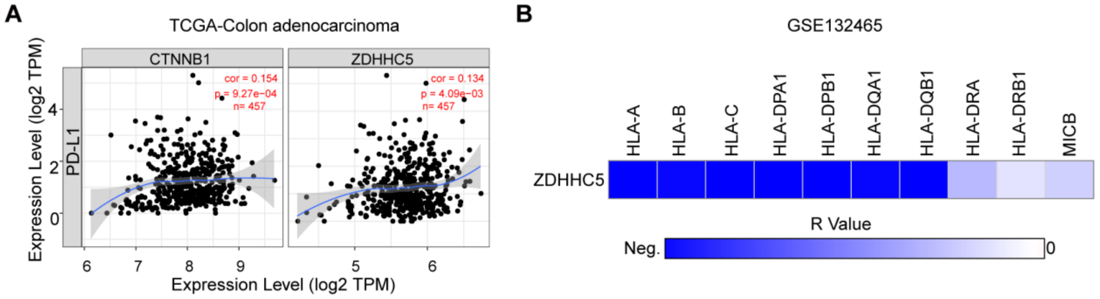

Figure S8 Association of *ZDHHC5* and *CTNNB1* with *PD-L1*, and ZDHHC5 with

antigen presentation gene expression. Related to Figure 5.

(A) *ZDHHC5* or *CTNNB1* mRNA expression levels are positively correlated with *PD-L1* mRNA expression level in colon adenocarcinoma from the TCGA database, based on data from the TIMER website (https://compbio.cn/timer1/).

(B) *ZDHHC5* expression in tumor epithelium is positively correlated with specific MHC gene expression in dendritic cells in the GSE132465 dataset.

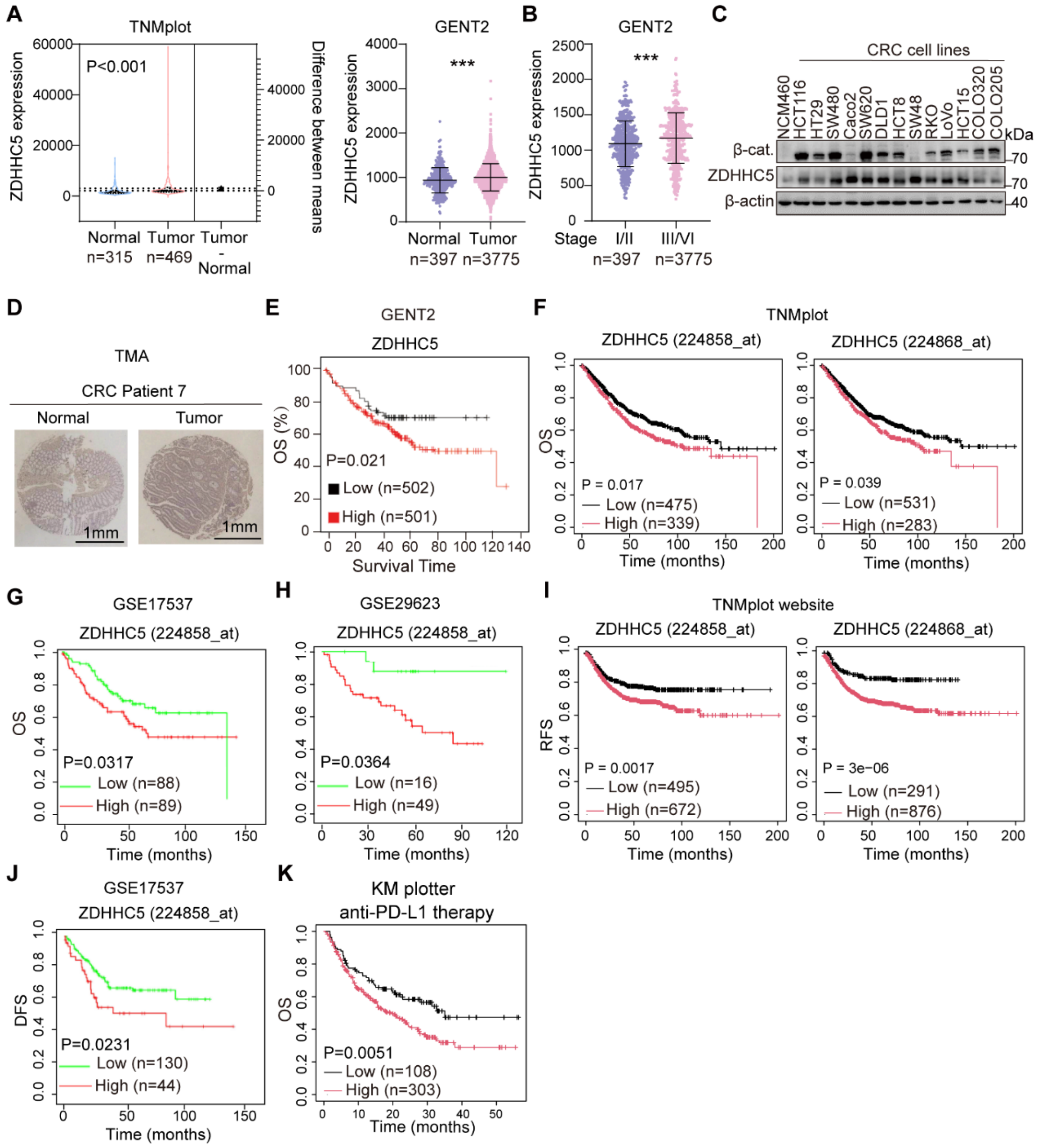

Figure S9 ZDHHC5 is highly expressed in CRC and correlates with poor prognosis. Related to Figure 6.

(A) *ZDHHC5* is highly expressed in CRC. Analysis of *ZDHHC5* expression in adjacent normal tissues and primary tumor tissues based on data from the TNMplot (https://tnmplot.com/analysis/) and GENT2 websites (http://gent2.appex.kr/gent2/).

(B) *ZDHHC5* expression is higher in advanced-stage CRC. Analysis of *ZDHHC5* expression in adjacent early-stage (Stage I/II) and advanced-stage (Stage III/IV) CRC tumor tissues based on the GENT2 website.

(C) ZDHHC5 is highly expressed in CRC tumor cells. Immunoblot analysis of ZDHHC5 in normal colon epithelial NCM460 and various CRC cell lines.

(D) Representative immunohistochemical staining of ZDHHC5 in CRC TMA using normal-tumor paired samples.

(E-J) High *ZDHHC5* expression correlates with poor prognosis in CRC. Kaplan-Meier survival curves comparing low versus high ZDHHC5 mRNA expression in human CRC patients, based on the GENT2, TNMplot, and GEO databases.

(K) High *ZDHHC5* expression was significantly associated with poorer OS in an independent cohort treated with anti-PD-L1 therapy, according to Kaplan-Meier plotter (https://kmplot.com/analysis/).

Data are presented as mean ± SD; For panels A and B, Student's t-test was used, with * $P < 0.05$, ** $P < 0.01$, *** $P < 0.001$. For panels E-K, the log-rank test was applied.

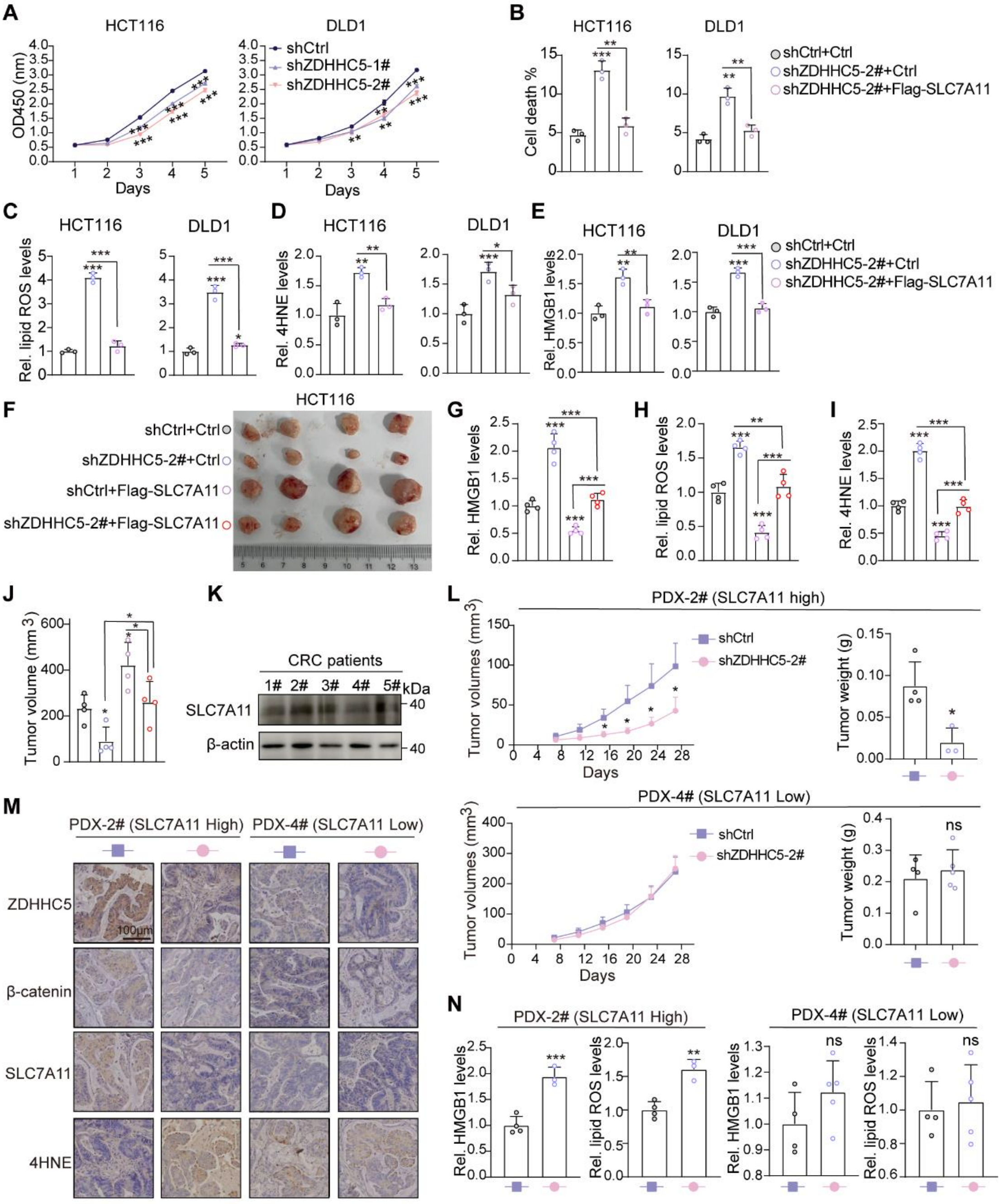

Figure S10 ZDHHC5 promotes resistance to immunogenic ferroptosis mediated by SLC7A11. Related to Figure 6.

(A) *ZDHHC5* knockdown inhibits CRC cell growth. Cell viability assays were performed in HCT116 and DLD1 cells with *ZDHHC5* knockdown.

(B-E) *ZDHHC5* knockdown promotes tumor cell death (**B**), increases lipid ROS levels (**C**), increases 4-HNE levels (**D**), and enhances extracellular HMGB1 release

(**E**), which can be rescued by SLC7A11 overexpression. SLC7A11 or control was overexpressed in HCT116 and DLD1 cells with *ZDHHC5* knockdown, followed by assessment of cell death rate using trypan blue staining, and detection of lipid ROS levels, 4-HNE levels, and extracellular HMGB1 release.

(F) *ZDHHC5* knockdown inhibits tumor growth, which is rescued by SLC7A11 overexpression. Images of subcutaneous tumors under various treatments. Xenograft experiments were conducted using HCT116 cells stably expressing the indicated vectors.

(G) Relative levels of HMGB1 in plasma from BALB/c nude mice bearing HCT116-derived xenografts shown in panel **F**, measured using a human HMGB1 ELISA kit.

(H-J) Lipid ROS levels (**H**), 4-HNE levels (**I**), and tumor volumes (**J**) were evaluated in the xenografts described in panel **F**.

(K) CRC Patient #2 showed high SLC7A11 protein expression, while CRC patient #4 showed low SLC7A11 expression. Immunoblot analysis of fresh tumor samples from five CRC patients.

(L) ZDHHC5 promotes tumor growth in an SLC7A11-dependent manner. PDXs with high or low SLC7A11 expression were treated with lentivirus expressing shZDHHC5, and tumors were harvested and weighed after treatment.

(M) Representative tumor IHC results in PDXs with high or low SLC7A11 expression. Scale bar: 100 μm.

(N) *ZDHHC5* knockdown increases human plasma HMGB1 levels in PDXs with high

SLC7A11 expression, but not in those with low SLC7A11 expression.

Data are presented as mean ± SD; Statistical significance was determined by Student's t test; * P < 0.05, ** P < 0.01, *** P < 0.001; "ns" stands for "not significant".

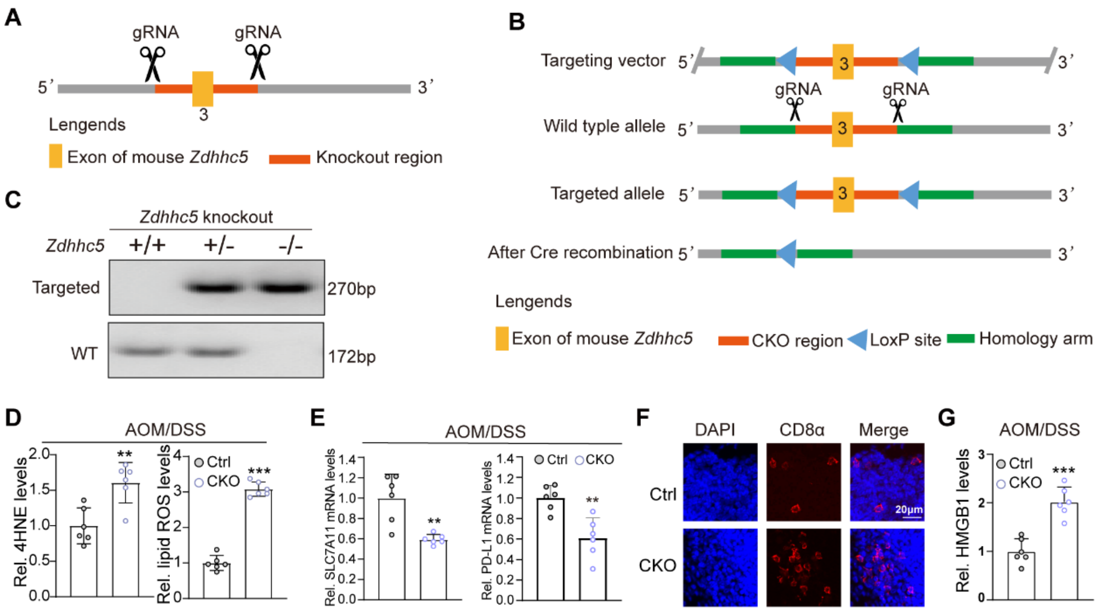

Figure S11 ZDHHC5 deficiency suppresses colorectal carcinogenesis by reversing immune evasion. Related to Figure 6.

(A) Schematic diagram of the construction of global *Zdhhc5* knockout mice.

(B) Schematic diagram of the construction of intestinal epithelium-specific Zdhhc5-knockout mice.

(C) Genotyping of mice by agarose gel electrophoresis.

(D) *Zdhhc5* ablation in mice increases 4-HNE and lipid ROS levels in CRC. AOM/DSS-induced CRC tumors in CKO mice were analyzed for 4-HNE and lipid ROS levels.

(E) *Zdhhc5* ablation in mice decreases *SLC7A11* and *PD-L1* expression in CRC. AOM/DSS-induced CRC tumors in CKO mice were analyzed by qPCR.

(F) *Zdhhc5* ablation in CKO mice promotes CD8$^+$ T cell infiltration in CRC. AOM/DSS-induced CRC tumors in Ctrl or CKO mice were subjected to immunofluorescence analysis.

(G) *Zdhhc5* ablation increases plasma HMGB1 levels in CRC. Plasma mouse HMGB1 levels were measured in AOM/DSS-induced CRC tumors from CKO mice.

Data are presented as mean ± SD; Statistical significance was determined by Student's t test; ** $P < 0.01$, *** $P < 0.001$.

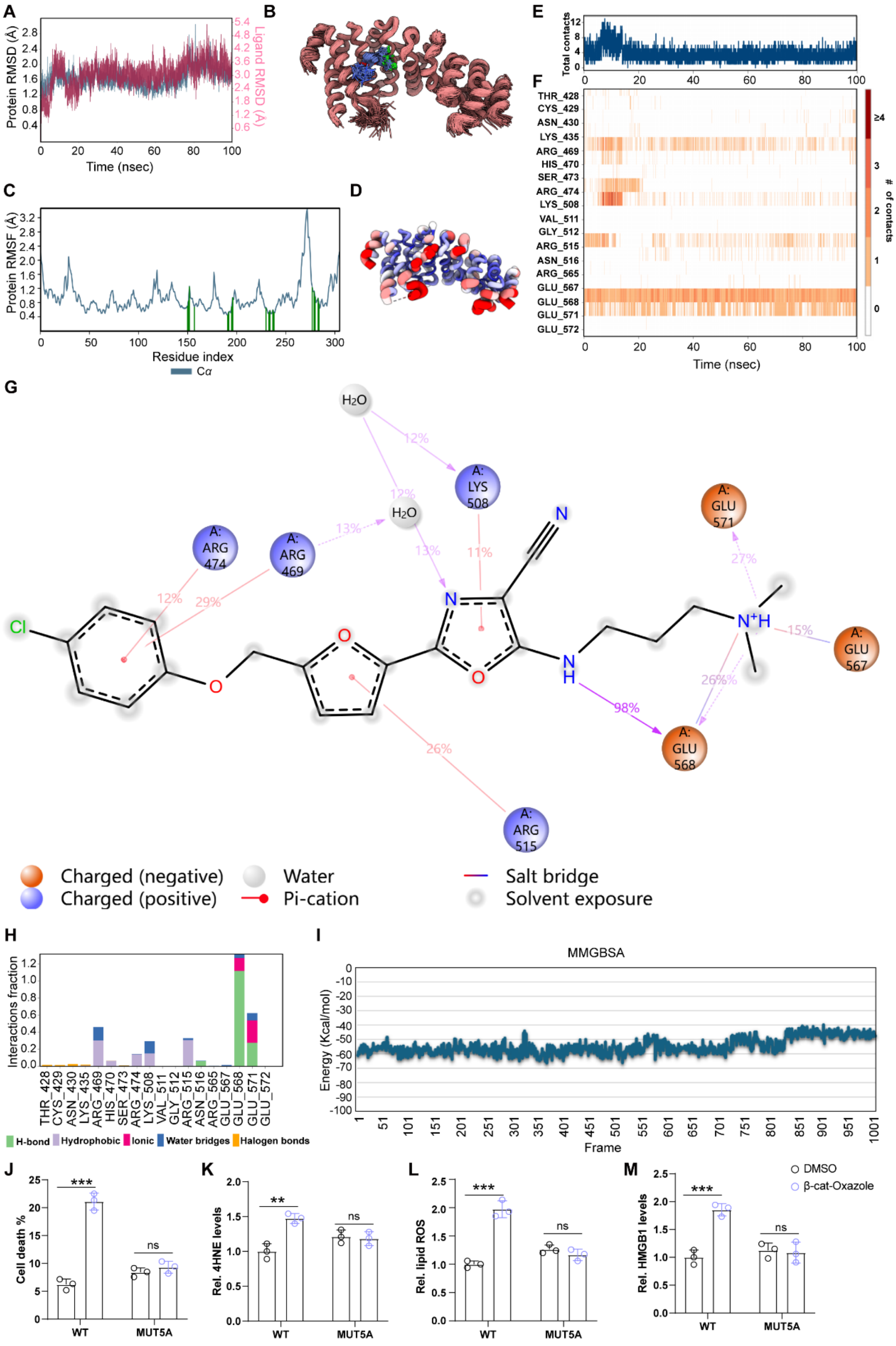

Figure S12 Molecular dynamics simulations and functional validation of the β-cat-Oxazole binding interface on β-catenin. Related to Figure 7.

(A) RMSD curve for β-catenin (blue) and β-cat-Oxazole (red) during the 100 ns molecular dynamics simulation.

(B) Superimposed structures of 100 conformations saved every 1 ns during the 100 ns molecular dynamics simulation.

(C) Residue-level RMSF analysis of β-catenin during the 20-100 ns molecular dynamics simulation. Green markers indicate amino acid residues interacting with β-cat-Oxazole.

(D) B-factor distribution of the β-catenin/β-cat-Oxazole complex based on MD trajectory analysis. The color reflects structural flexibility: red regions have high B-factors (indicating high flexibility, corresponding to large RMSF), while blue regions have low B-factors (indicating rigid structures with small RMSF).

(E) Total number of interactions between β-cat-Oxazole and β-catenin over the course of the molecular dynamics simulation.

(F) Changes in the contact frequency of key interaction residues between β-cat-Oxazole and β-catenin over time during the molecular dynamics simulation.

(G) Interaction network analysis of key residues at the β-catenin binding site and β-cat-Oxazole, showing types of interactions (e.g., salt bridges, Pi-cation), and the interaction frequency of amino acids (e.g., ARG, GLU) and water molecules.

(H) Analysis of the types and proportions of interactions between key residues at the β-catenin binding site and β-cat-Oxazole, displaying contributions from hydrogen bonds, hydrophobic interactions, and other interaction modes.

(I) Fluctuation of MMGBSA binding free energy of the β-cat-Oxazole/β-catenin

complex during the final 10 ns of the simulation (1001 frames).

(J-M) Functional validation of the predicted binding interface in *CTNNB1*-knockout HCT116 cells reconstituted with WT β-catenin or a binding-deficient quintuple mutant (Arg469A, Lys508A, Arg515A, Glu568A, and Glu571A, referred to as MUT5A). Cells were treated with β-cat-Oxazole (20 μM) or DMSO. Cell death was measured by trypan blue staining assays (**J**). Relative mRNA levels of immunogenic ferroptosis markers, including 4-HNE (**K**), lipid ROS levels (**L**), and HMGB1 release (**M**) were also evaluated.

Data are presented as mean ± SD; Statistical significance was determined by Student's t test; ** P < 0.01, *** P < 0.001; "ns" stands for "not significant".

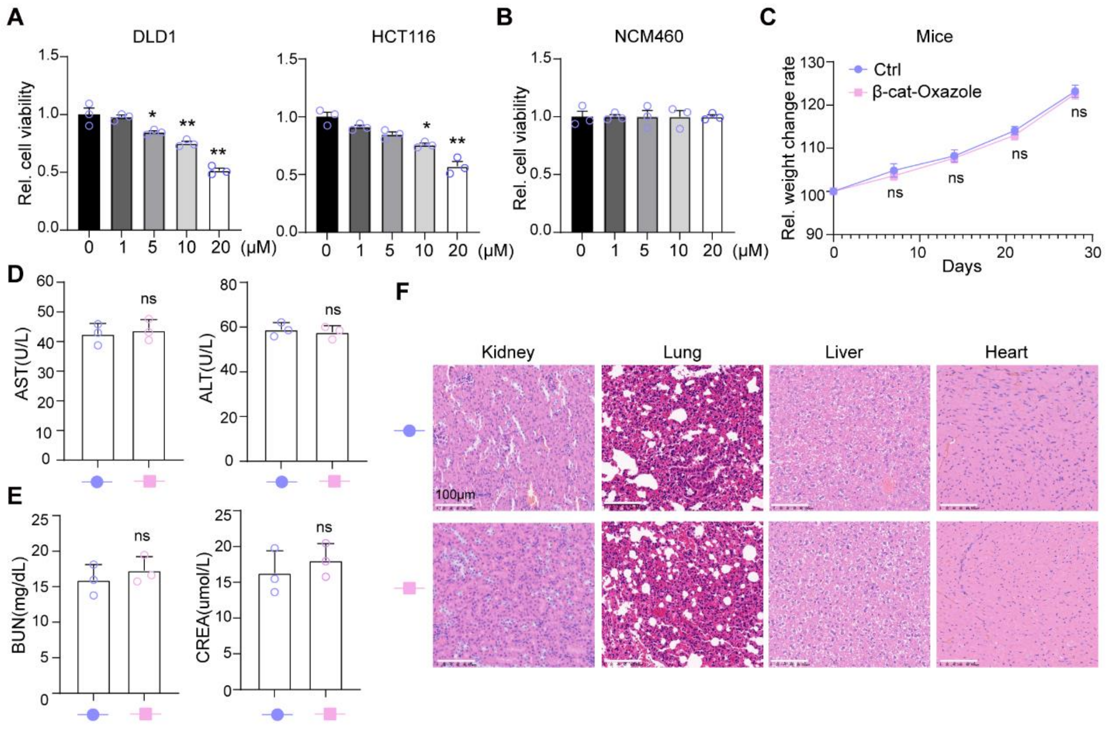

Figure S13 Evaluation of β-cat-Oxazole safety in vitro and in vivo. Related to Figure 7.

(A and B) β-cat-Oxazole treatment reduces cell viability in CRC cells, but not in normal cells. Cell viability of HCT116 and DLD1 cells (**A**), and the normal human colon epithelial cell line NCM460 (**B**), following a 3-hour treatment with the indicated concentrations of β-cat-Oxazole.

(C) Body weight monitoring of mice during β-cat-Oxazole administration.

(D) Serum levels of ALT and AST.

(E) Serum levels of CREA and BUN.

(F) Representative histopathological analysis of the major organs. Scale bar, 100 μM.

Data are presented as mean ± SD; Statistical significance was determined by Student's t test; ** $P < 0.01$; "ns" stands for "not significant".

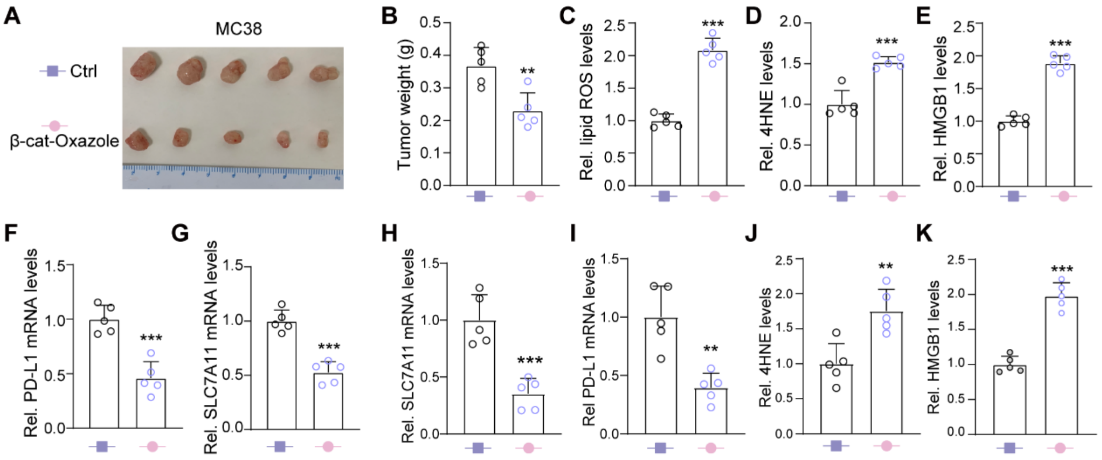

Figure S14 β-cat-Oxazole suppresses tumor growth reactivates antitumor immunity in MC38 subcutaneous and AOM/DSS-induced CRC models. Related to Figure 7.

(A) β-cat-Oxazole treatment inhibits MC38 subcutaneous tumor growth. photographs of subcutaneous MC38 tumors harvested from C57BL/6 mice treated with vehicle

(Ctrl) or β-cat-Oxazole (20 mg/kg, i.p.)

(B-D) Quantitative analysis of the MC38 subcutaneous tumor model. Tumor weight (**B**), lipid ROS levels (**C**) and 4-HNE levels (**D**) were evaluated in the xenografts described in panel **A**.

(E) Relative levels of HMGB1 in plasma from C57BL/6 mice bearing MC38-derived xenografts shown in panel **A**, measured using a mouse HMGB1 ELISA kit.

(F and G) qPCR analysis of *PD-L1* (**F**) and *SLC7A11* (**G**) expression was evaluated in the xenografts described in panel A.

(H and I) β-cat-Oxazole treatment significantly reduces *SLC7A11* (**H**) and *PD-L1* (**I**) expression in mouse CRC. AOM/DSS-induced CRC tumors in mice treated with β-cat-Oxazole were analyzed by qPCR.

(J) β-cat-Oxazole treatment significantly increases 4-HNE levels in mouse CRC. 4-HNE levels in AOM/DSS-induced mouse CRC tumors from β-cat-Oxazole-treated or control mice were measured by ELISA.

(K) Relative levels of HMGB1 in plasma from C57BL/6 mice bearing MC38-derived xenografts, measured using a mouse HMGB1 ELISA kit.

Data are presented as mean ± SD; Statistical significance was determined by Student's t test; ** $P < 0.05$, ** $P < 0.01$, *** $P < 0.001$.
